# Films of linear conjugated polymer as photoanodes for oxidation reactions†

**DOI:** 10.1039/d4sc03512g

**Published:** 2024-08-13

**Authors:** Shuming Chai, Shun Zhao, Jiaxin Su, Jinshui Zhang, Xiong Chen, Reiner Sebastian Sprick, Yuanxing Fang

**Affiliations:** a State Key Laboratory of Photocatalysis on Energy and Environment, College of Chemistry, Fuzhou University 350002 P. R. China chenxiong987@fzu.edu.cn yxfang@fzu.edu.cn; b Department of Pure and Applied Chemistry, University of Strathclyde Glasgow G1 1XL UK sebastian.sprick@strath.ac.uk; c Sino-UK International Joint Laboratory on Photocatalysis for Clean Energy and Advanced Chemical & Materials, Fuzhou University Fuzhou 350002 P. R. China

## Abstract

Photoelectrochemical (PEC) devices hold huge potential to convert solar energy into chemical energy. However, the high cost of raw materials and film processing has hindered its practical use. In this study, we attempt to tackle this issue by fabricating straightforward semiconducting polymer films. These films function as photoanodes for various oxidation reactions, including water oxidation and oxidative organosynthesis. The structures of the polymer were assessed by incorporating electron-rich and electron-deficient co-monomers into dibenzo[*b*,*d*]thiophene sulfone materials. Furthermore, to gain comprehensive insight into the performance, we conducted both steady-state and *in operando* investigations, revealing that the active site on the polymer surface determines the rate of the conversion process. This study marks a significant stride towards leveraging economically viable semiconductors in PEC systems for efficient solar-to-chemical conversions. It addresses the challenges of high material costs and complex film processing, paving the way for the scaled-up application of this burgeoning technology.

## Abstract 2

Photoelectrochemical (PEC) devices hold huge potential to convert solar energy into chemical energy.

## Graphical abstract

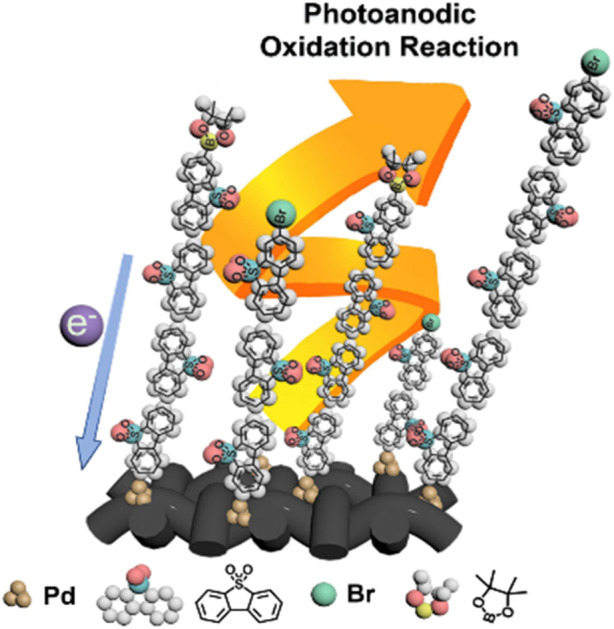

## Introduction

Photoelectrochemical (PEC) water splitting has triggered a surge in research efforts within the field of photocatalysis. This encompasses studies on photocatalysts,^1–3^ study of underlying mechanisms,^4,5^ development of systems,^6,7^ and exploration of applications.^8–10^ Significantly, these studies have facilitated the discovery of numerous promising potential applications aimed at addressing energy and environmental challenges. These applications include hydrogen production through water splitting,^11^ degradation of organic pollutants,^12^ organic transformations,^13^ and many others applications.^14,15^ In the context of water splitting, PEC systems still stand out as the most efficient ones for converting solar energy into chemical energy compared to suspension based photocatalytic systems.^16,17^ This superior efficiency can be largely attributed to the applied voltage bias, which enhances the separation and transfer of photoexcited charges, while also reducing charge carrier recombination.^18–20^ Nevertheless, it is important to note that the construction of PEC systems is often associated with high costs, primarily due to expensive materials and film processing, which have limited its practical applications.

Metal-free photocatalysts have gained prominence since the initial discovery of carbon nitride polymers' activity for water-splitting.^21–23^ Building upon this breakthrough, a wide array of organic semiconductors has been developed as photocatalysts, including linear polymers,^24,25^ covalent organic frameworks,^26–31^ triazine-based frameworks,^32–35^ and many others.^36–40^ These materials, composed solely of Earth-abundant elements, hold the potential to supplement inorganic counterparts, paving the way for cost-effective PEC systems.^41–45^ Notably, polymers, in contrast to inorganic materials, offer the advantage that they can be processed into films controlling their morphologies on micro- and nano-scale. More importantly, the semiconductor properties of these materials can be finely manipulated through the incorporation of different building blocks and synthetic approaches.^46^ As an example, the integration of donor–acceptor (D–A) motifs was demonstrated tailoring of semiconducting polymers, facilitating the dissociation of the photo-excited excitons and thereby enhancing their performance for photoredox reactions.^25,47–50^ To date, most efforts in the realm of organic-based PEC systems have predominantly focused on developing photoanodes based on carbon nitride materials.^7,51–53^ While there have been significant advancements in this area, there remains a compelling need to diversify the range of organic semiconductors, with a particular emphasis on creating materials that deliver chemically stable and active responsiveness under visible light irradiation.^54^

In this study, we synthesized six linear conjugated polymer films on carbon cloth (CC) and subsequently employed these as photoanodes. A typical photoanode, a dibenzo[*b*,*d*]thiophene sulfone (FSO) monomer, is co-polymerized with an additional electron-rich or electron-deficient co-monomer, *i.e.*, phenylene, pyridylene, pyrrolylene, and tetrafluorobenzene, to form D–A motifs. Additionally, the homopolymer of FSO was prepared, which was found to exhibit the highest performance for water oxidation. Subsequently, this FSO photoanode was further used in oxidative organosynthesis. We were able to use the photoanode for two model reactions; specifically, the synthesis of *N*-benzylidenebenzylamine through the oxidation of benzylamine and the synthesis of methyl phenyl sulfoxide *via* the oxidation of methyl phenyl sulfide, and the corresponding selectivity was realized up to 92% and 99%, respectively, presenting advantages over photocatalytic power system.

Steady-state and *in operando* measurements were performed to establish structure–property relationships between the polymer structure and their performance in photoanodic reactions. In these systems, the active site determines the rate for this conversion: through the measurements, we established that the FSO photoanode is efficient in accumulating of photoexcited charges on its sulfone group, resulting in the best performance for oxidation reactions. This work serves as a proof-of-concept study for employing cost-effective polymer semiconductors to construct a PEC system through conventional synthesis. Furthermore, it highlights strategic approach for designing polymer structures, thereby improving solar-to-chemical conversion as well as the selectivity and yield in organic synthesis.

## Results and discussion

**Fig. 1:**
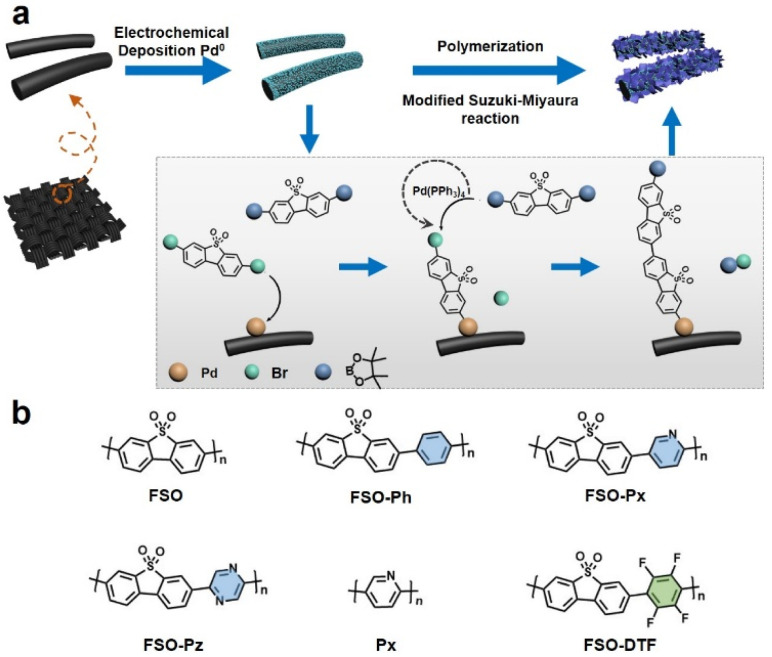
(a) Proposed mechanism of the growth procedure of the linear conjugated polymer photocatalysts on carbon cloth *via* a modified Suzuki–Miyaura reaction. (b) Chemical structures of the linear semiconductor polymers used in this study.

The approach to growth linear conjugated polymers on a CC substrate is shown in Fig. 1. Metallic palladium nanoparticles were electrochemically deposited onto the surface of the CC (Fig. S1†), which act as initiation sites for the polymerization. Based on X-ray photoelectron spectroscopy (XPS) results (Fig. S2†), the nanoparticles were observed to be physically anchored on the CC. Under Suzuki–Miyaura reaction conditions, the dibromo monomer can bond to the palladium nanoparticles through an oxidative addition reaction. This bonded monomer on the surface of CC can then act as the active site for further polymerization by a modified Pd(0)-catalyzed Suzuki–Miyaura reaction. Giving photoanodes that have the linear polymers were anchored on the CC. Six photoanodes consisting of linear polymers on CC, were made, FSO, FSO-Ph, FSO-Px, FSO-Pz, Px, and FSO-DTF, the corresponding structures are presented in Fig. 1b.

The design of these polymer structures was based on rational considerations stemming from the encouraging photocatalytic activity of FSO, which, in its powdered form, has demonstrated excellent performance in water oxidation reactions.^55^

Variations in the polymer structure were achieved by introducing co-monomer units into the FSO system, including phenyl-, pyridyl-, or pyrrolyl- as electron donors, or tetrafluorobenzene- as electron withdrawing moieties, aiming at optimizing the strength of the D–A effect in the polymer system. Additionally, Px-n was synthesized as a reference material.

**Fig. 2:**
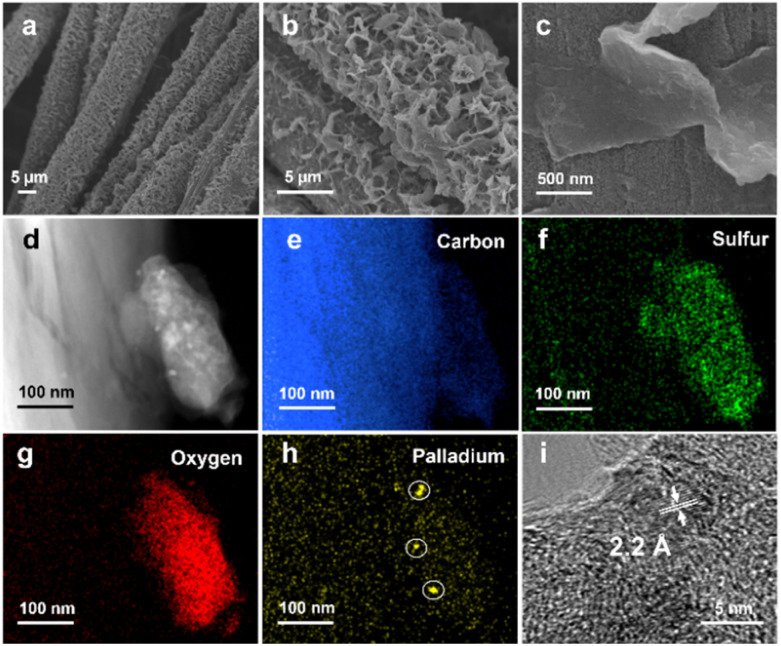
SEM images to present (a) the morphology of the photoanode, (b) and (c) the morphology of the FSO nanosheets. TEM images illustrate (d) a piece of FSO on CC, along with the elemental mapping of (e) carbon, (f) sulfur, (g) oxygen, and (h) palladium on this piece. (i) The lattice fringes of Pd between the carbon cloth and conjugated polymers.

The linear polymers can be discerned on the surface of the CC (Fig. S3–S7†), and an FSO photoanode is shown as a typical example in Fig. 2. In the scanning electronic microscopy (SEM) images (Fig. 2a), the polymers appeared as nanosheets (Fig. 2b), exhibiting a uniform distribution across the CC surface. Obviously, these nanosheets were grown on the CC surface, with parts of the sheets oriented vertically relative to the surface (Fig. 2c). This vertical orientation of the nanosheets potentially increases the available active sites for surface redox reactions. Furthermore, transmission electron microscopy (TEM) images of the FSO nanosheet are presented in Fig. 2d. These images show the presence of elements, including carbon (Fig. 2e), sulfur (Fig. 2f), and oxygen (Fig. 2g), which were evenly distributed within the FSO sheet, and the FSO material and the CC are clearly identifiable.

In Fig. 2h, Pd nanodots were clearly observed at the interface between the CC and FSO, with lattice fringes exhibiting a distance of *ca.* 2.2 Å, corresponding to the (111) facet of metallic Pd (Fig. 2i). Given the vital role of Pd as a catalyst in the *in situ* growth of linear polymer photoanodes, control experiments were conducted to grow the linear conjugated polymers on CC substrates without Pd loading (Fig. S8†). In these experiments, the resulting polymer featured a bulky appearance and appeared physically attached to the CC, resembling films formed by drop-coating polymer powder onto the CC (Fig. S9†). This underscores the importance of Pd for facilitating *in situ* growth. Further optimization was carried out by adjusting the electrodeposition times, the quantities of [Pd(PPh_3_)_4_], and time of Suzuki–Miyaura reaction. SEM images have illustrated the morphology of the photoanodes (Fig. S10 to S12†).

**Fig. 3:**
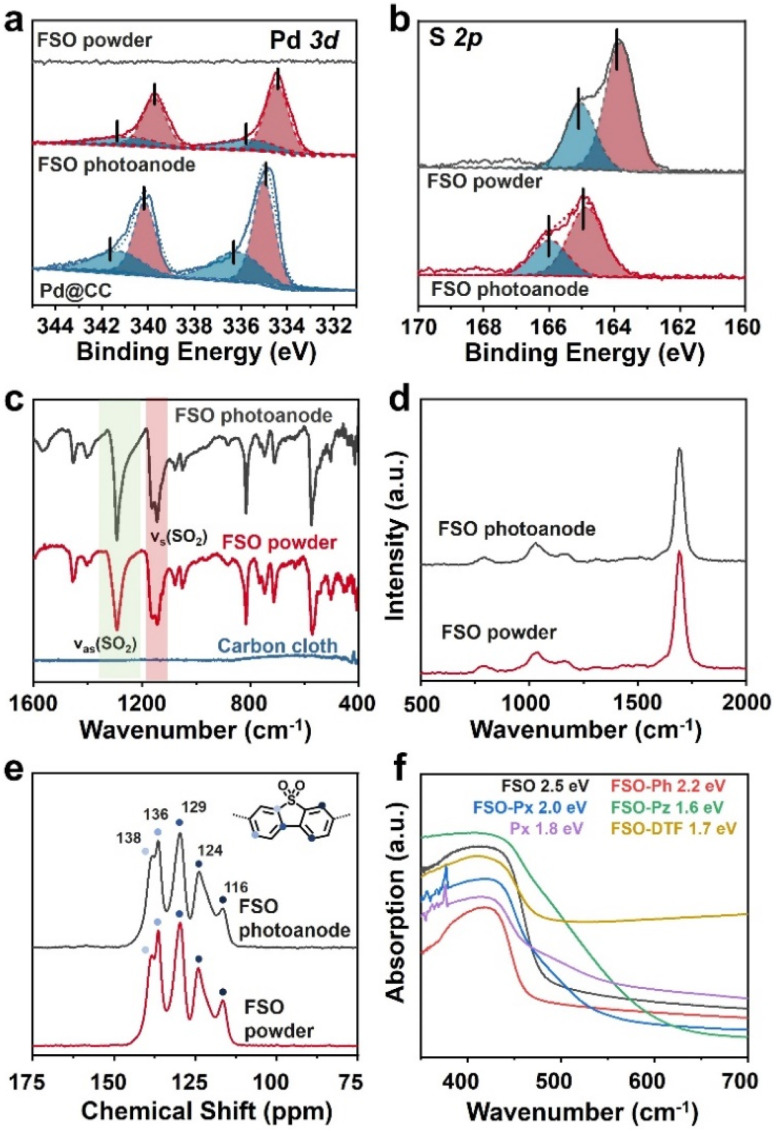
(a) Pd 3d and (b) S 2p XPS spectra of FSO. (c) FTIR spectra, (d) Raman spectra, and (e) ^13^C CP/MAS NMR chemical shift of FSO. (f) UV/vis DRS of the linear polymer based photoanodes.

XPS analysis was conducted to probe the presence of Pd and S in FSO. In the Pd 3d spectra as shown in Fig. 3a, the peaks at 337.5 eV and 332.2 eV were assigned to the Pd 3d5/2 and Pd 3d3/2 orbitals, respectively. When FSO nanosheets were formed on the Pd particles, the binding energies of the peaks shifted to 336.9 eV and 331.7 eV, respectively. Meanwhile, the S 2p spectra in Fig. 3b disclosed the binding energies of 165.02 eV and 163.83 eV, corresponding to the S 2p3/2 and S 2p1/2 orbitals, respectively. Notably, the *in situ*-grown polymer films displayed increased binding energies for the S 2p orbital when compared to powdered FSO. These shifts in binding energies were indicative of electronic interactions among the linear polymer, the Pd particles, and the CC substrate, highlighting potential chemical binding between them.

Further investigation into the chemical properties of the linear polymer-based photoanode was conducted by Fourier-transform infrared spectroscopy (FTIR), Raman spectroscopy, and solid-state ^13^C cross polarization/magic angle spinning nuclear magnetic resonance spectroscopy (^13^C CP/MAS NMR), and comparisons were made with their powdered counterparts as reference samples. In the FTIR spectra of FSO, as shown in Fig. 3c, prominent bands at wavenumbers of 1292 and 1141 cm^−1^ were attributed to stretching vibration of the sulfone group.^56^ The sulfone group in FSO-Ph, FSO-Px, FSO-Pz, and FSO-DTF was also identified in their respective spectra, indicating the formation of the polymer structures (Fig. S13–S17†). In the case of Px, bands at 1580 cm^−1^ were assigned to the wavenumber of pyridine cyclic groups,^57^ which agrees with FSO-Px. The Raman spectra of FSO were presented in Fig. 3d and others were shown in Fig. S18–S22.† For FSO-Ph, FSO-Px, FSO-Pz, and FSO-DTF, a band at 1149 cm^−1^ was observed indicating the presence of sulfone groups.^58^ For Px and FSO-Px, a band at 1249 cm^−1^ was observed, respectively, and both were associated with the stretching vibration of the pyridine ring.^59^ Thus, both FTIR and Raman confirm the formation of the linear polymer on CC.

^13^C CP/MAS NMR chemical shifts of FSO were presented in Fig. 3e, and the peaks at the chemical shifts of 116, 124, 129, 136, and 138 ppm were observed, attributing to the carbon atoms in benzene skeleton of dibenzo[*b*,*d*]thiophene sulfone. The results agree with the literature reports.^60^ The peaks of all the carbon atoms from the benzene skeleton were also observed for FSO-Ph, FSO-Px, FSO-Pz, and FSO-DTF, indicating the formation of the corresponding chemical structures (Fig. S23–S27†). For Px and FSO-Px, the characteristic peak at 150.9 ppm corresponds to pyridine.^61^ Moreover, from the FTIR, Raman, and ^13^C CP/MAS NMR spectra, the chemical structures of the polymers in powder form were found to be comparable to those on the photoanodes.

The optical properties of the polymer photoanodes were investigated by ultraviolet-visible diffuse reflectance spectroscopy (UV/vis DRS) as shown in Fig. 3f. Generally, these polymer photoanodes exhibited the capability to absorb visible light. Band-gaps of FSO, FSO-Ph, FSO-Px, FSO-Pz, Px, and FSO-DTF were calculated using Tauc plots (Fig. S28†), giving values of 2.5, 2.2, 2.0, 1.6, 1.8, and 1.7 eV, respectively.

**Fig. 4:**
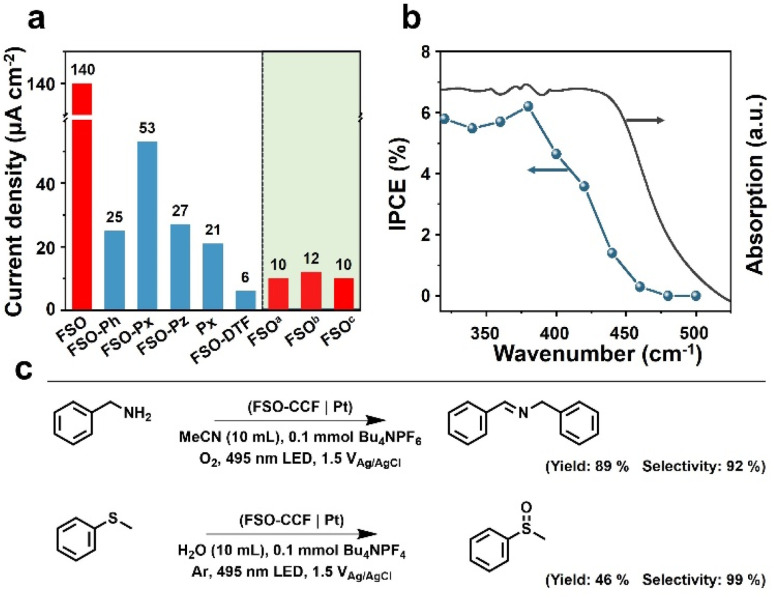
(a) Photocurrent densities of the linear polymer photoanodes for water oxidation reaction. (b) Incident photon-to-current conversion efficiency for water oxidation reaction and light absorption spectrum of FSO photoanode. (c) The yield and selectivity of benzylamine oxidation and thioanisole oxidation by optimal FSO photoanode.

The polymer photoanodes were used as working electrodes for oxidation reactions in a three-electrode configuration, with Pt and Ag/AgCl acting as the cathode, and reference electrode, respectively. Oxygen evolution reaction (OER) through water oxidation was tested initially. Linear sweep voltammetry was performed by illumination from a sunlight simulator (Fig. S29– S32†), and transient photocurrent densities were recorded at the voltage bias of 1.23 V *versus* reversible hydrogen electrode (V_RHE_ as optimal performance shown in Fig. 4a) (Fig. S33–S39†). These results were obtained after adjusting the synthesis conditions (Fig. S40–S43†). FSO exhibited the highest photocurrent density of all polymer photoanodes prepared through *in situ* synthesis, at 140 μA cm^−2^, whereas FSO-DTF presented the lowest photocurrent density at 6 μA cm^−2^ (Fig. 4a).

Additionally, three reference photoanodes were prepared for comparison (Fig. 4a): ^a^FSO was prepared by drop-casting FSO powder on fluorine-doped tin oxide glass, ^b^FSO was prepared by drop-casting as-prepared FSO powder onto CC, and ^c^FSO was obtained by growing FSO on CC without loading any additional Pd. Notably, the photocurrent density of these FSO counterparts photoanodes was lower than 12 μA cm^−2^. These values were an order of magnitude lower than the FSO photoanode which was prepared by *in situ* growth. This finding underscores the critical role of the chemical bond between the photocatalytic films and the conductive electrode in the PEC system.

The water oxidation reaction was performed for *ca.* 50 min (Fig. S44†), and the photocurrent density was generally preserved. The photoanode after this reaction was also studied by SEM (Fig. S45†), and the morphology was generally maintained, revealing the excellent stability of this photoanode for water oxidation reaction. The rates of the hydrogen evolution reaction were also measured (Fig. S46†), yielding a photocurrent density of 22 μmol cm^−2^ h^−1^. When 10% triethanolamine was used as a sacrificial agent, the rate increased to 28 μmol cm^−2^ h^−1^. Incident photon-to-current conversion efficiency (IPCE) was acquired for FSO photoanode, and the results are shown in Fig. 4b. The observed photocurrent responses generally match the absorption spectrum (Fig. 3f). Remarkably, visible light with the wavelength up to 470 nm was capable of driving OER. The highest IPCE value, reaching 8.5%, was achieved at a wavelength of 400 nm. The results show the potential of polymer photoanodes for solar to chemical conversion.

Following this, the optimised FSO photoanode was then also used for organic oxidation reactions in a proof-of-concept study. Two typical examples are shown in Fig. 4c, including *N*-benzylidenebenzylamine synthesis by benzylamine oxidation and methyl phenyl sulfoxide synthesis by oxidation of methyl phenyl sulfide.^62,63^ These reactions represent important functional group transformations in organic chemistry since both imide and sulfoxide are vital building blocks for fine chemicals and pharmaceuticals.^64–66^ Compared to powder systems, the transformations use no sacrificial reagents and are accompanied by hydrogen evolution. As a result, the yields for *N*-benzylidene benzylamine and methyl phenyl sulfoxide were determined to be 89% and 46%, with very high selectivities of 92% and 99%, respectively. These results show the potential advantages of PEC system for organic transformations over powder-based systems (Table S1†).

**Fig. 5:**
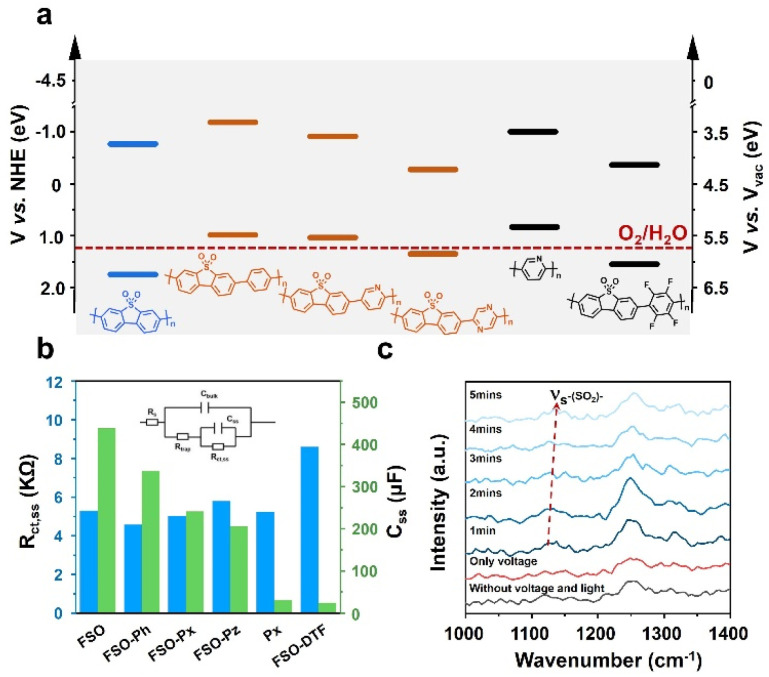
(a) Bandgap of the linear semiconductor polymers. (b) The *R*_ct,ss_ and *C*_ss_ of the linear polymer based photoanode obtained from Nyquist plot of EIS (inset: the equivalent circuit for EIS). (c) Time-resolved *in situ* Raman spectra of the FSO photoanode for water oxidation reaction.

To understand the performance of the linear conjugated polymer-based photoanodes, their physicochemical properties were analyzed. The conduction band of the polymer photoanodes was determined using Mott–Schottky plots derived from electrochemical impedance spectroscopy (EIS) (Fig. S47†). The band structures were subsequently determined by aligning these results with bandgap values (Fig. 3f), as illustrated in Fig. 5a. The introduction of the motifs in FSO highly affects their band structures. With respect to pristine FSO, the introduction of electron-donor or electron-withdrawing motifs decreases the bandgap. The incorporation of electron-donating motifs, such as phenyl-, pyridyl-, and pyrrolyl-groups, lowers both the highest occupied molecular orbital (HOMO) and the lowest unoccupied molecular orbital (LUMO) levels. However, the effect of the band positions was found to be less significant to affect the PEC performance.

Photoluminescence spectroscopy (PL) was employed to study the exciton binding energies (Fig. S48†). For the FSO, the introduction of the electron-rich motifs generally causes a reduction in binding energy (Fig. S49†). Contrarily, in this case, the presence of an electron-withdrawing group in FSO leads to an increase in the binding energy of the photo-exciton. Surprisingly, these results contradict the outcomes of photocurrent density measurements. To further understand the dynamics of photoexcitation, EIS was conducted, and its Nyquist plots are shown in Fig. S50 (Tables S3 and S4†). The analysis focused on the low-frequency resistance (*R*_ct,ss_) and low-frequency capacitance (*C*_ss_), as shown in Fig. 5b. In this system, *R*_ct,ss_ is associated with the separation and transfer of photoexcited charges for the reaction, while *C*_ss_ is linked to the capacity of charges to accumulate on the active site, highly relevant to surface reactions. It is evident that when an external bias is applied to the linear polymer photoanode, *R*_ct,ss_ values were similar across different photoanodes, indicating that the formation of D–A structure for the polymer is ineffective in improving charge dynamics in the PEC system. Conversely, the *C*_ss_ of the photoanode followed a similar trend to the photocurrent density, suggesting that surface reaction is a key factor in determining the performance. Among the various photoanodes, FSO exhibited the strongest capability for accumulating photoexcited charges on the active site, consequently facilitating oxidation reactions.

To gain insight into activity of the surface reaction, an *in situ* Raman analysis was performed on the polymer photoanodes while the water oxidation reaction took place (Fig. S51†). The magnified time-resolved spectra, depicted in Fig. 5c, highlight key features. The peak at 1129 cm^−1^ corresponds to the reverse symmetric stretching vibration of the sulfone group, while the peak at approximately 1250 cm^−1^ is attributed to the C–C stretching vibration in the benzene ring.^58^ It is noteworthy that the sulfone group is only visible during simultaneous application of both electric potential and irradiation. Crucially, the wavenumber of this sulfone group gradually increases as the water oxidation reaction progresses. In contrast, the wavenumber associated with the C–C stretching vibration remains generally consistent throughout the reaction. This observation points to the likelihood that the sulfone group is specifically responsive to this oxidation reaction. We note that the potential accumulation of photoexcited charges on the sulfone group emerges as a crucial determinant of the oxidation reaction rate. Conversely, the formation of a D–A structure on the photoanode leads to a dispersion of charges, thereby diminishing the reaction rate.

Among the photoanodes studied, FSO exhibited the highest photocurrent density. The results can be attributed to its suitable valence band potential for water oxidation and, more importantly, its superior capability to accumulate photoexcited charges at the active site. Compared to polymers with different molecular structures, the efficiency of the active site is considered crucial in designing new photoanodes for photoelectrochemical water oxidation, rather than focusing solely on the donor–acceptor (D–A) structure design. In particular, we identified that the sulfone group plays a pivotal role in the oxidation reaction through *in situ* Raman analysis, providing guidance for designing highly efficient photoanodes. Therefore, we conclude that optimizing the bandgap and increasing the active site, specifically the sulfone group, can enhance the performance of water oxidation.

## Conclusions

In conclusion, linear polymer-based films were fabricated on CC through a modified Suzuki–Miyaura reaction, and were subsequently employed as photoanodes for oxidation reactions. A typical photoanode, FSO, was polymerized, and additionally, electron-rich and electron-deficient co-monomers such as phenyl, pyridyl, pyrrolyl, and tetrafluorobenzene groups were introduced to form D–A motifs. The FSO-based photoanode emerged as the system with the highest performance in the water oxidation reaction and was also effective in other oxidative organic synthesis. Furthermore, we carried out studies to understand the relationship between polymer structure and its performance in photoanodic reactions. We found that the active site on the polymer surface determines the rate of the conversion process; *i.e.* in the FSO photoanode, photoexcited charges accumulated on the sulfone group, but the introduction of a co-monomer into the polymer resulted in a dispersion of photoexcited charges, reducing the reaction rate. The proof-of-concept study shows that polymer semiconductors can be used to potentially construct cost- and performance-effective PEC systems. It also shows that the design of conjugated polymers for solar-to-chemical conversions offers new opportunities, which are yet to be exploited.

## Experimental

All synthesis procedures and a detailed overview of the analytical instrumentation and characterization methods are provided in the ESI.† A summary of the central compounds and reaction conditions is provided here.

### The preparation of Pd-CC

Pd particles were loaded onto a carbon cloth (CC) by electrodeposition. The CC (10 mm × 20 mm) electrode was sequentially cleaned by ultrasonication with acetone, ethanol, and ultrapure water for 10 min before it was blow-dried. The electrolyte solutions were 0.1 mmol KCl solution containing 0.5 mmol of H_2_PdCl_4_ solution. Detail of the preparation: an aqueous solution of *n*(PdCl_2_) : *n*(HCl) = 1 : 2 (pH value ≈ 6.0) in 100 mL H_2_O, and the solution was reddish-brown in color. Before the electrochemical experiment, high-purity N_2_ gas was blown into the electrolytic cell for 20 min to remove the oxygen in the solution; during the experiment, high-purity N_2_ gas was introduced above the solution to prevent the oxygen in the air from dissolving in the electrolyte. All experiments were carried out at room temperature.

The Pd-CC anode was performed using a standard three-electrode electrochemical cell attached to a BioLogic VSP-300 electrochemical workstation. The CC electrode is used directly as the working photoanode. The Pt foil (10 mm × 10 mm) and Ag/AgCl electrode were used as the counter electrode and the reference electrode, respectively. The proportion of CC immersed into the electrolyte is 1 cm^2^ and the electrodeposition method was constant voltage with −0.2 V (*vs.* V_Ag/AgCl_) and the electrodeposition time is 1 min.

### Preparation of linear polymer powder

General procedure for Suzuki–Miyaura-type polycondensations. A flask was charged with monomers, DMF, an aqueous solution of K_2_CO_3_ (2 M), and [Pd(PPh_3_)_4_]. The mixture was heated at 423 K in an Ar atmosphere for two days. Upon cooling to room temperature, the precipitate were collected by vacuum filtration and rinsed with H_2_O and acetone, respectively, followed by further purification through Soxhlet extraction with tetrahydrofuran for 1 day. The polymer powders were then dried under a vacuum. FSO-n^55^ was synthesized using previously reported procedures.

### Synthesis of FSO-Ph

3,7-Dibromodibenzo[*b*,*d*]thiophene sulfone (749 mg, 2 mmol), 1,4-phenylenediboronic acid (332 mg, 2 mmol), K_2_CO_3_ (8 mL, 2 M), [Pd(PPh_3_)_4_] (40 mg), DMF (40 mL) were used in this reaction. The product was obtained as a green powder (362 mg, yield 33%).

### Synthesis of FSO-Px

2,5-Dibromopyridine (472 mg, 2 mmol), dibenzo[*b*,*d*]thiophene, 3,7-bis(4,4,5,5-tetramethyl-1,3,2-dioxaborolan-2-yl)dibenzo[*b*,*d*]thiophene sulfone (749 mg, 2 mmol), K_2_CO_3_ (8 mL, 2 M), [Pd(PPh_3_)_4_] (40 mg), DMF (40 mL) were used in this reaction. The product was obtained as a yellow powder (492 mg, yield 35%).

### Synthesis of FSO-Pz

3,7-Bis(4,4,5,5-tetramethyl-1,3,2-dioxaborolan-2-yl)dibenzo[*b*,*d*]thiophene sulfone (749 mg, 2 mmol), 2,5-dibromopyrazine (476 mg, 2 mmol), K_2_CO_3_ (8 mL, 2 M), [Pd(PPh_3_)_4_] (40 mg), DMF (40 mL) were used in this reaction. The product was obtained as a green powder (570 mg, yield 46%).

### Synthesis of Px

5-Bromopyridin-2-ylboronic acid (404 mg, 2 mmol), K_2_CO_3_ (8 mL, 2 M), [Pd(PPh_3_)_4_] (40 mg), DMF (40 mL) were used in this reaction. The product was obtained as a green powder (160 mg, yield 40%).

### Synthesis of FSO-DTF

1,4-Dibromo-2,3,5,6-tetrachlorobenzene (747 mg, 2 mmol), 3,7-bis(4,4,5,5-tetramethyl-1,3,2-dioxaborolan-2-yl)dibenzo[*b*,*d*]thiophene sulfone (749 mg, 2 mmol), K_2_CO_3_ (8 mL, 2 M), [Pd(PPh_3_)_4_] (40 mg), DMF (40 mL) were used in this reaction. The product was obtained as a green powder (344 mg, yield 23%).

### Preparation of linear polymer photoanode

General procedure for modified Suzuki–Miyaura-type polycondensations. Before the reaction, the prepared Pd-CC anode was put into the reaction vessel and mixed with the reactants. After 2 days of reaction, the powder product was filtered and collected, whilst the polymer photoanodes electrode was cleaned using ultrasonication in acetone and water for 10 min each.

### Synthesis of FSO photoanode

3,7-Dibromodibenzo[*b*,*d*]thiophene sulfone (281 mg, 0.75 mmol), 3,7-bis(4,4,5,5-tetramethyl-1,3,2-dioxaborolan-2-yl)dibenzo[*b*,*d*]thiophene sulfone (351 mg, 0.75 mmol), K_2_CO_3_ (4 mL, 2 M), [Pd(PPh_3_)_4_] (1 mg), DMF (40 mL) were used in this reaction.

### Synthesis of FSO-Ph photoanode

3,7-Dibromodibenzo[*b*,*d*]thiophene sulfone (281 mg, 0.75 mmol), 1,4-phenylenediboronic acid (124.3 mg, 0.75 mmol), K_2_CO_3_ (4 mL, 2 M), [Pd(PPh_3_)_4_] (1 mg), DMF (40 mL) were used in this reaction.

### Synthesis of FSO-Px photoanode

2,5-Dibromopyridine (177.7 mg, 0.75 mmol), 3,7-bis(4,4,5,5-tetramethyl-1,3,2-dioxaborolan-2-yl) dibenzo[*b*,*d*]thiophene sulfone (351 mg, 0.75 mmol), K_2_CO_3_ (4 mL, 2 M), [Pd(PPh_3_)_4_] (1 mg), DMF (40 mL) were used in this reaction.

### Synthesis of FSO-Pz photoanode

3,7-Bis(4,4,5,5-tetramethyl-1,3,2-dioxaborolan-2-yl)dibenzo[*b*,*d*]thiophene sulfone (351 mg, 0.75 mmol), 2,5-dibromopyrazine (178.5 mg, 0.75 mmol), K_2_CO_3_ (4 mL, 2 M), [Pd(PPh_3_)_4_] (1 mg), DMF (40 mL) were used in this reaction.

### Synthesis of Px photoanode

5-Bromopyridin-2-ylboronic acid (423.7 mg, 1.5 mmol), K_2_CO_3_ (4 mL, 2 M), [Pd(PPh_3_)_4_] (1 mg), DMF (40 mL) were used in this reaction.

### Synthesis of FSO-DTF photoanode

3,7-Bis(4,4,5,5-tetramethyl-1,3,2-dioxaborolan-2-yl)dibenzo[*b*,*d*]thiophene sulfone (351 mg, 0.75 mmol), 1,4-dibromo-2,3,5,6-tetrachlorobenzene (280 mg, 0.75 mmol), K_2_CO_3_ (4 mL, 2 M), [Pd(PPh_3_)_4_] (1 mg), DMF (40 mL) were used in this reaction.

### Photoelectric experiments

The photocurrent, linear sweep voltammetry, and electrochemical impedance spectroscopy were studied on an electrochemical workstation (VSP-300, Bio-Logic) using a three-electrode system with an Ag/AgCl electrode, a platinum plate, and the polymer photoanode acting as the reference electrode, counter electrode, and work electrode, respectively. The electrolyte solution was 0.1 mol NaOH. A light source equipped with an AM 1.5G filter (100 mW cm^−2^, LCS-100, Newport) was used as in the experiments. The frequency range of electrochemical impedance was 200 kHz to 100 mHz.

### Photoelectrochemical oxidation reaction experiments

The PEC study was performed in an undivided cell and conducted in a three-electrode system, with an Ag/AgCl electrode, a platinum plate, and FSO photoanode being used as the reference electrode, counter electrode, and work electrode, respectively.

### Benzylamine oxidation experiments

Benzylamine (0.1 mmol, 10 μL), MeCN (10 mL), and tetrabutylammonium hexafluorophosphate (0.1 mmol, 38.7 mg) were added to undivided cell and then O_2_ was purged into the system. The reaction was initiated with a 495 nm LED continuous agitating with stirring. The reaction time was 1 hour. The quantity of products was detected by GC with anisole as an internal standard.

### Thioanisole oxidation experiments

Thioanisole (0.25 mmoL, 30 μL), H_2_O (10 mL), and tetrabutylammonium tetrafluorophosphate (0.1 mmol, 32.9 mg) were added to undivided cell, and then Ar was purged into the system. The reaction was illuminated with a 495 nm LED with continuous agitating with stirring. The reaction time was 1 hour. The quantity of products was detected by GC with chlorobenzene as the internal standard.

## Data availability

All relevant data is available in the manuscript and as part of the ESI.†

## Author contributions

S. Chai, S. Zhao, J. Su and Y. Fang were performed experiments and data curation. J. Zhang and R. S. Sprick revised the manuscript. X. Chen and Y. Fang conceived the project, funding acquisition, and writing of the original draft.

## Conflicts of interest

There are no conflicts to declare.

## Supplementary Material

SC-OLF-D4SC03512G-s001
