# Supplementary material for: Films of linear conjugated polymer as photoanodes for oxidation reactions
Source: Chem Sci. 2024 Aug 13;15(37):15496–503. doi: 10.1039/d4sc03512g (PMC11376065; doi:10.1039/d4sc03512g)
Supplement: SC-015-D4SC03512G-s001 [file SC-015-D4SC03512G-s001.pdf]

Supplementary Information (SI) for Chemical Science.
This journal is © The Royal Society of Chemistry 2024

Electronic Supplementary Information

Experimental Section

Materials. Dibenzo[*b,d*]thiophene sulfone, *N*-bromosuccinimide (NBS), bis(pinacolato)diboron, [1,1-bis(diphenylphosphine)ferrocene]dichloropalladium(II), H_2PdCl_4, 1,4-phenylenediboronic acid, $[Pd(PPh_3)_4]$, 2,5-dibromopyridine, 2,5-dibromopyrazine, 5-bromopyridin-2-ylboronic acid, 1,4-dibromo-2,3,5,6-tetrachlorobenzene, triphenylphosphine, benzylamine, thioanisole, tetrabutylammonium hexafluorophosphate, tetrabutylammonium tetrafluorophosphate, and palladium(II) chloride were purchased from Innochem Co. Ltd. (Beijing, China). Sulfuric acid, chloroform, potassium acetate, ethyl acetate, acetonitrile, ethyl alcohol, potassium chloride, hydrochloric acid, dimethyl sulfoxide, anhydrous ethanol, anhydrous diethyl ether, *N, N*-dimethylformamide (DMF), acetonitrile, and potassium carbonate (K_2CO_3) were purchased from Sinopharm Chemical Reagent Co. Ltd. (Shanghai, China). 3,7-Dibromodibenzo[*b*,*d*]thiophene sulfone[1] and 3,7-bis(4,4,5,5-tetramethyl-1,3,2-dioxaborolan-2-yl)dibenzo[*b*,*d*]thiophene sulfone[2] were prepared according to the reported procedures.

Characterizations. Fourier transforms infrared (FTIR) spectra were recorded on a Nicolet (thermos Nicolet Nexus) 670 FTIR spectrometer. Raman spectra were conducted by LabRAM Odyssey (HORIBA France SAS). UV-Vis Diffuse reflectance spectroscopy (UV/Vis DRS) was performed on a Varian Cary 500 Scan UV-visible system. X-ray photoelectron spectroscopy (XPS) was performed using a Thermo ESCALAB 250 instrument with a monochromatized Al Kα line source (200 W), and C 1s binding energy (284.8 eV) was used as the reference. The morphology was investigated by scanning electron microscopy (SEM, Jsm-6700 F) and transmission electron microscopy (TEM, an FEI Tencai 20 microscope). Solid-state ^{13}C cross polarization/magic angle spinning nuclear magnetic resonance (^{13}C CP/MAS NMR) spectroscopy was recorded on a Bruker Advance III 500 spectrometer. The photoelectrocatalytic performance analysis was determined on a VSP-300 BioLogic electrochemical station with three electrode systems. Photoluminescence (PL) spectra were measured on a Hitachi F-7000 FL spectrophotometer with an emission spectrum acquired by the excitation wavelength of 400 nm. Gas chromatography (GC) analysis was performed on a Nexis GC-2030 (SHIMADZU Japan) detecting the photoelectric oxidation reaction products.

IPEC is calculated according to Equation 1.

$$IPCE\ (\%) = 1240 \times I/(\lambda P_{input})\ \text{(Equation 1)}$$

where λ and P_{input} is the wavelength and power (mW/cm^2) of the incident light (Xenon lamp), respectively, and I is photocurrent density.

The bandgap is calculated by Kubelka-Munk plots.

$$(F(R)E)^{1/n} = B(E-E_g)\ \text{(Equation 2)}$$

where R represents reflectivity and E equals hν (where h is Planck's constant and ν is the light frequency), and n equals 2. Plotting (F(R)E)1/n against E yields a linear equation, with the X-axis intercept representing the value of bandgap.

Morphological characterization

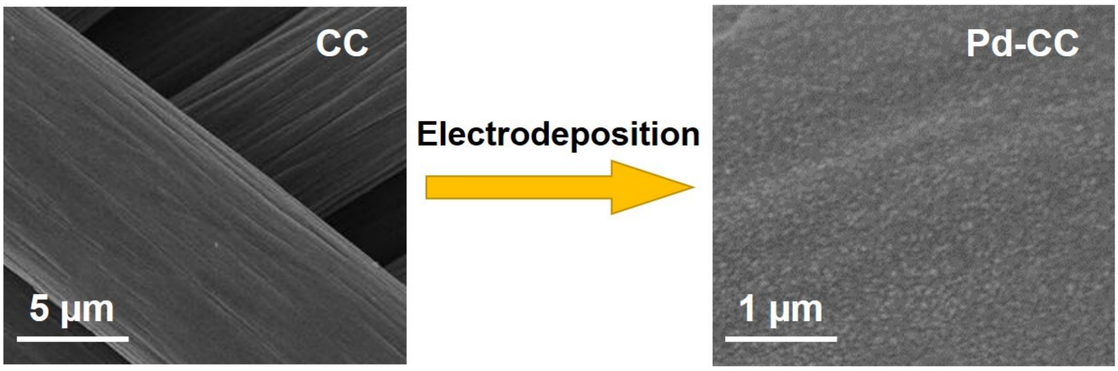

Fig. S1 The picture of electrodeposition Pd on carbon cloth.

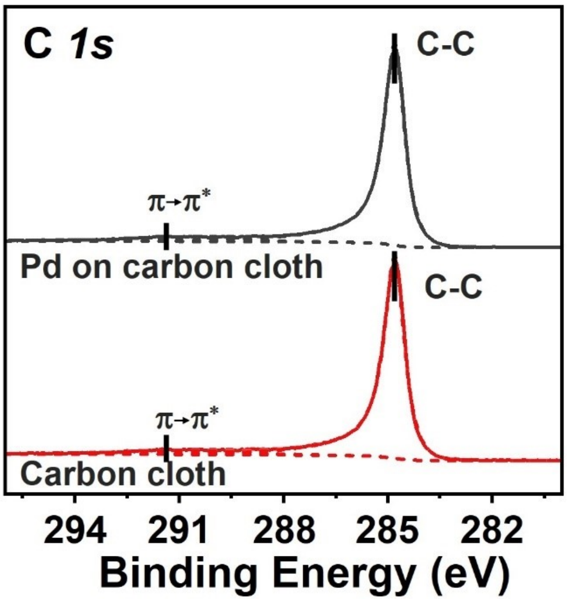

Fig. S2 C 1s XPS spectra of carbon cloth and Pd on carbon cloth. The C 1s XPS spectra exhibited two peaks at binding energies of 284.8 eV and 292.2 eV, corresponding to C-C

and π→π* transitions.[3,4] The preserved C 1s spectrum after Pd loading indicated no chemical interaction between Pd and the carbon cloth. Electrochemical loading resulted in strong adhesion through physical interaction between Pt and the carbon cloth.

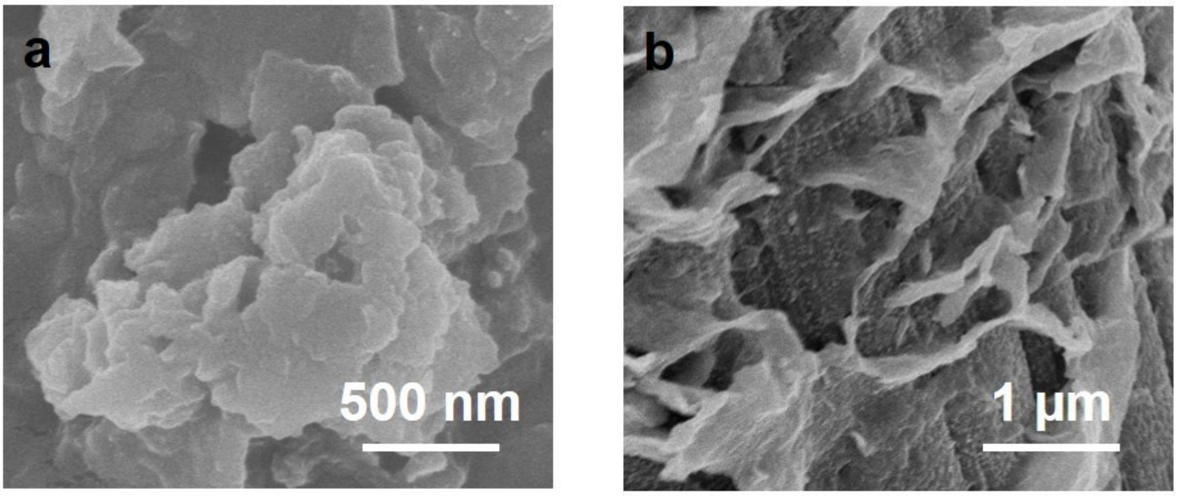

Fig. S3 SEM images of (a) FSO-Ph powder and (b) FSO-Ph grown on the Pd-CC as photoanode.

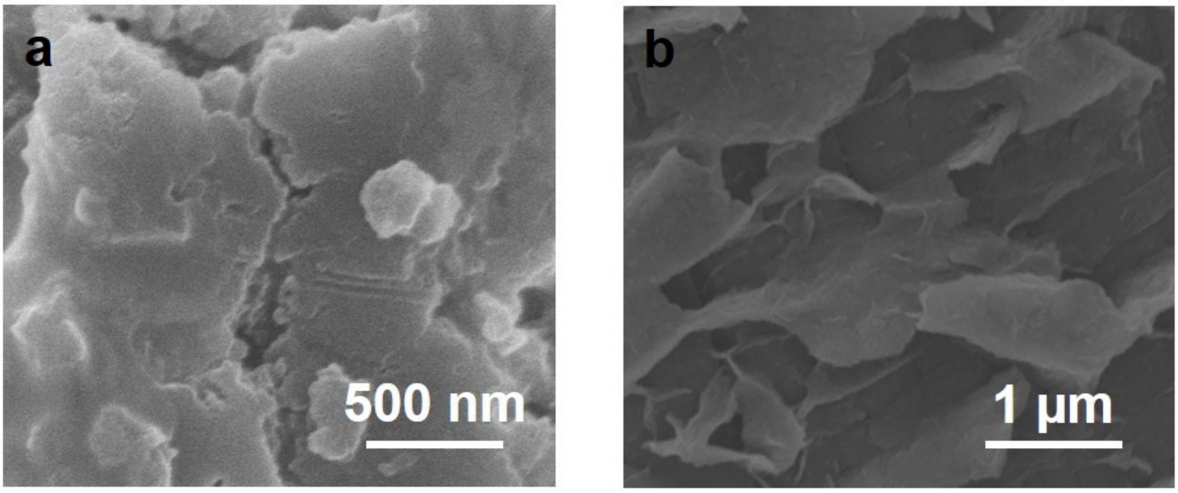

Fig. S4 SEM images of (a) FSO-Px powder and (b) FSO-Px grown on the Pd-CC as photoanode.

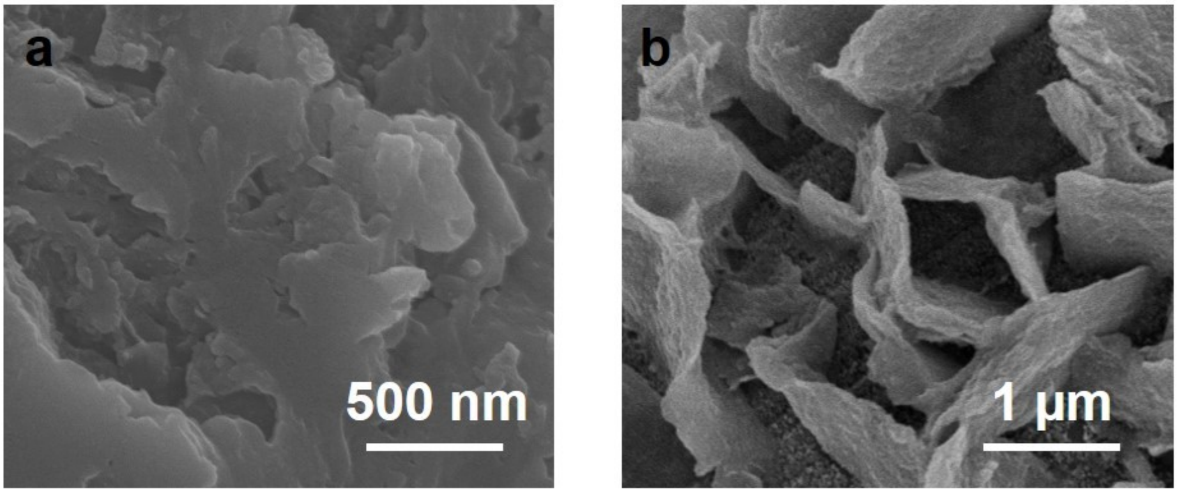

Fig. S5 SEM images of (a) FSO-Pz powder and (b) FSO-Pz grown on the Pd-CC as photoanode.

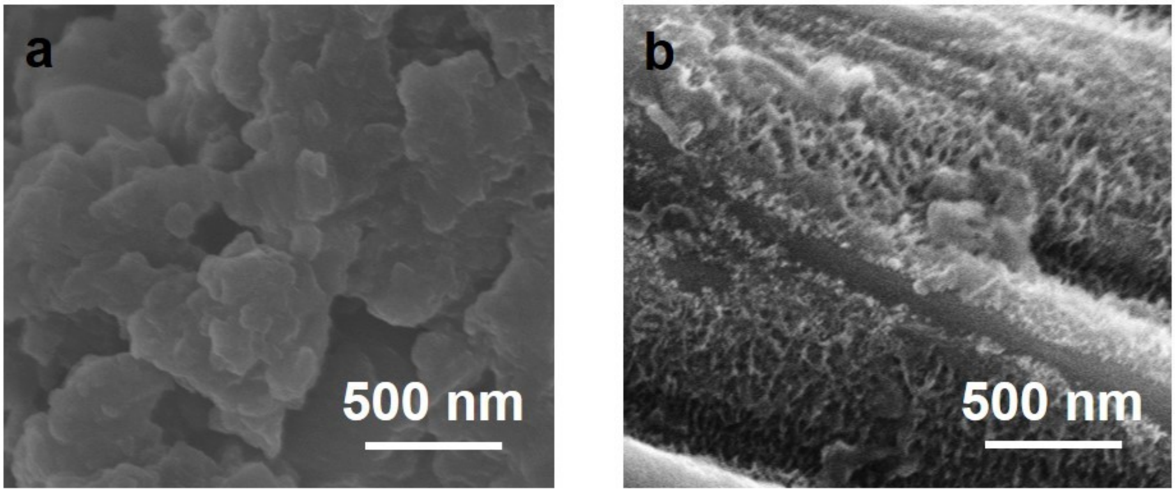

Fig. S6 SEM images of (a) Px powder and (b) Px grown on the Pd-CC as photoanode.

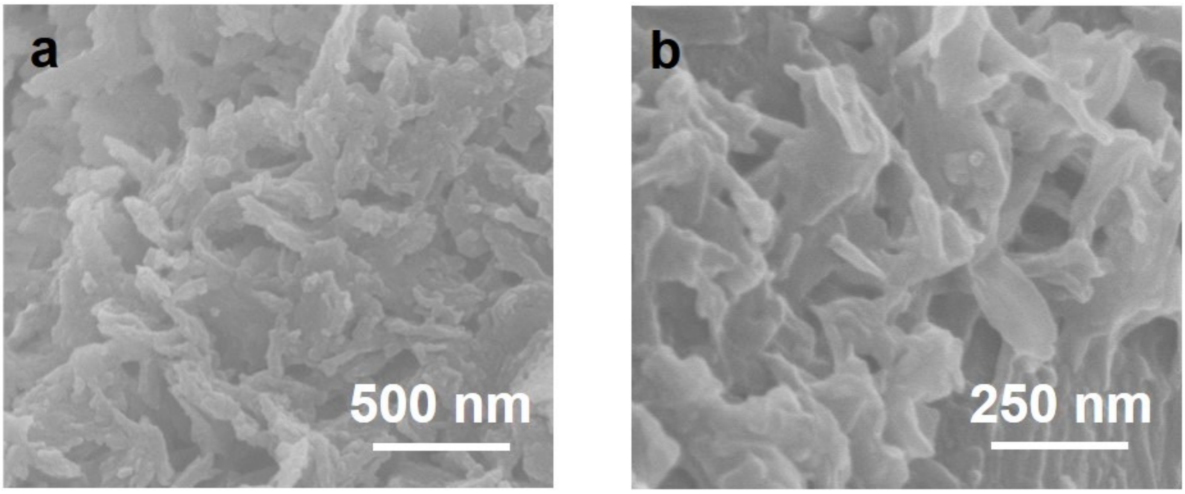

Fig. S7 SEM images of (a) FSO-DTF powder and (b) FSO-DTF grown on the Pd-CC as photoanode.

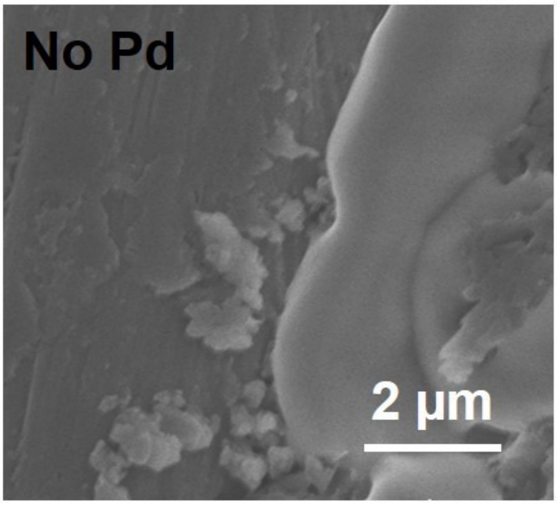

Fig. S8 SEM image of FSO photoanode without electrodeposited Pd. We cannot observed as a symmetrical distribution lamellar structure of polymer when Pd was absent on CC. When the polymer formed as a lamellar accumulation structure on CC, the barrier of charge transfer between the polymer and carbon cloth can be reduced.

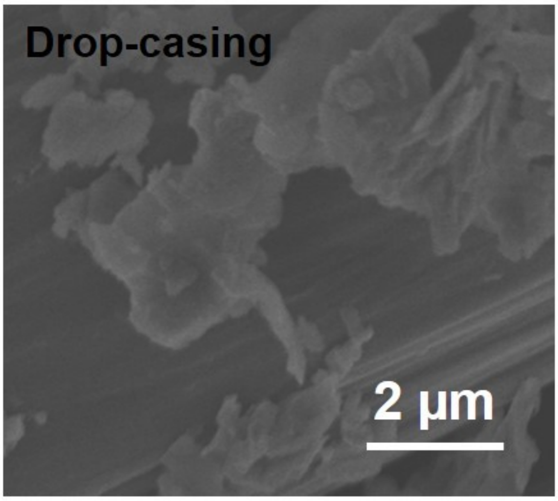

Fig. S9 SEM images of FSO photoanode prepared by drop-casing. It is observed that the polymer powder exists on the surface of the carbon cloth in the form of bulk accumulation. The poor adhesion generates numerous defects which appear to reduce the charge transfer efficiency between the polymer and the carbon fiber.

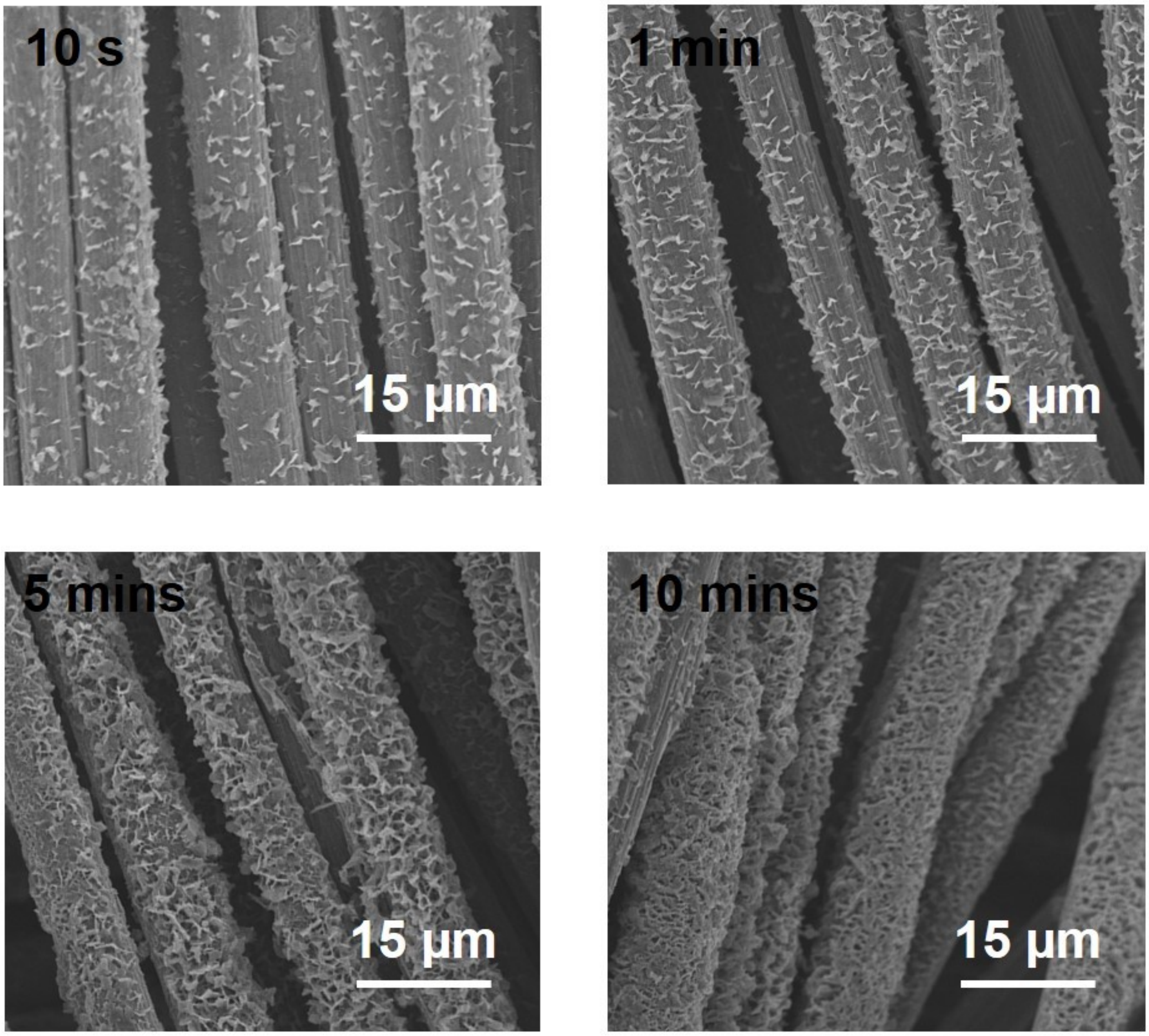

Fig. S10 SEM images of FSO photoanodes after different electrodeposition times. The polymer had a symmetrical distribution lamellar structure on the CC when the electrodeposition was 1 minute. The shorter the Pd electrodeposition, the less distribution content of polymer on the CC. However, when the time of electrodeposition is too long, it leads to excess growth of polymer on the CC, resulting in poorer distribution of the material covering more space which results in accumulation of the polymer on the CC.

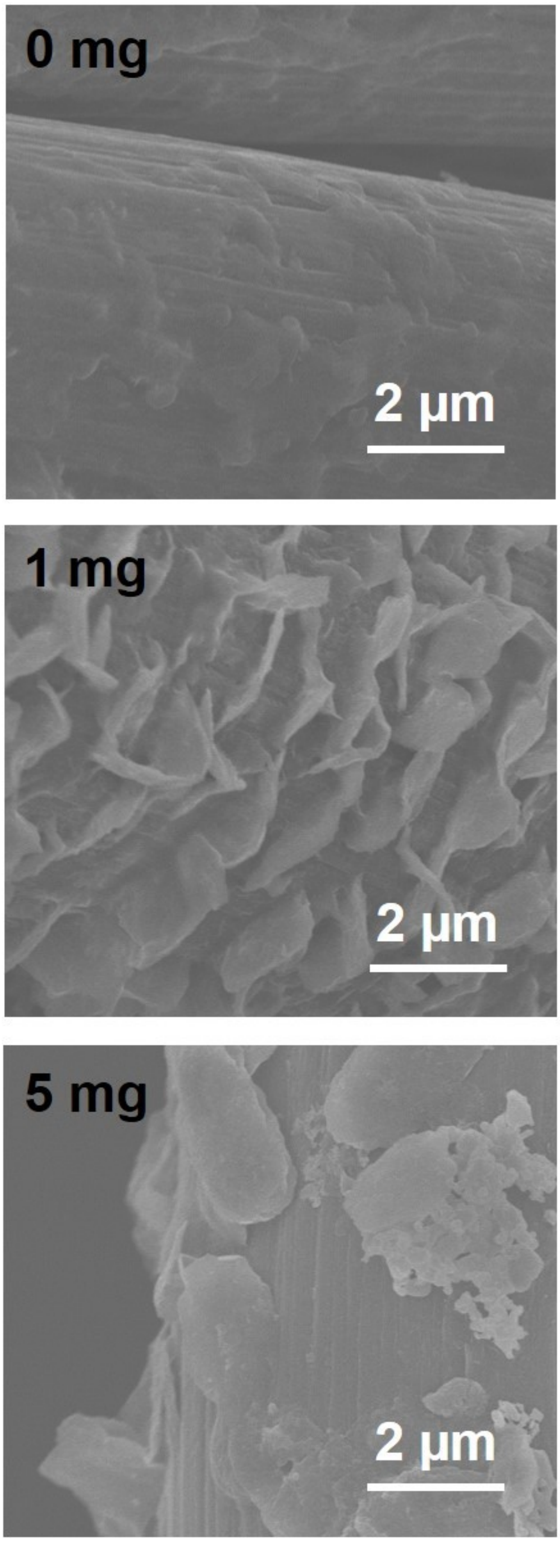

Fig. S11 SEM images of FSO photoanodes produced using different quantities of [Pd(PPh_3)$_4$] in the Suzuki-Miyaura cross-coupling reaction.

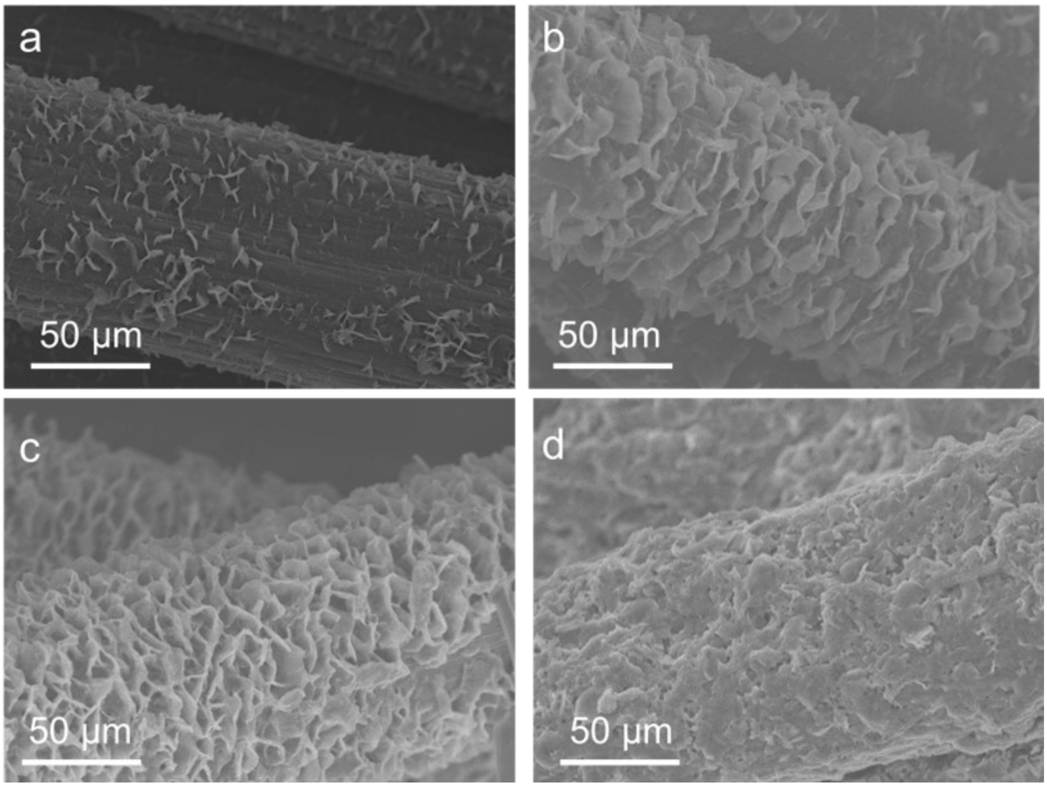

Fig. S12 SEM images of FSO photoanodes produced using different Suzuki-Miyaura reaction time.

Structural characterization

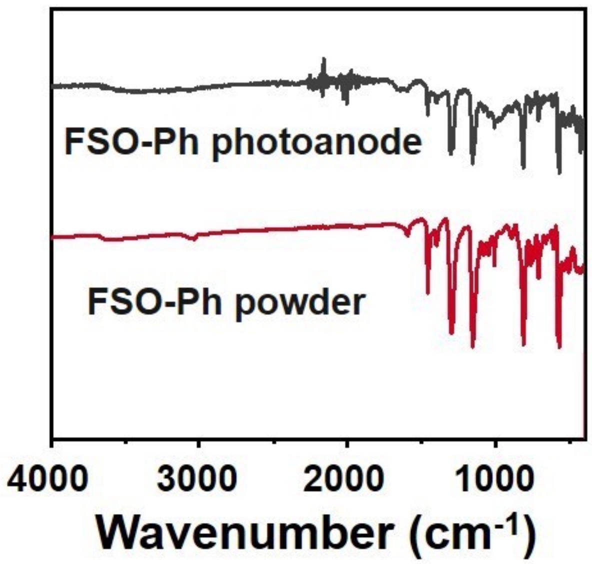

Fig. S13 FTIR spectra of FSO-Ph photoanode and powder.

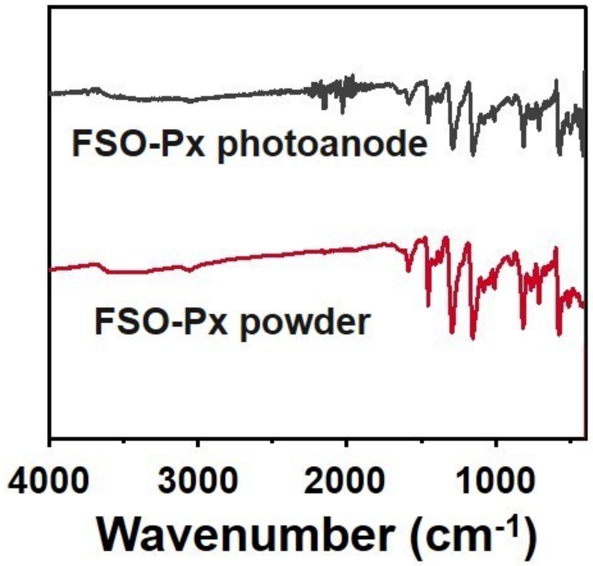

Fig. S14 FTIR spectra of FSO-Px photoanode and powder.

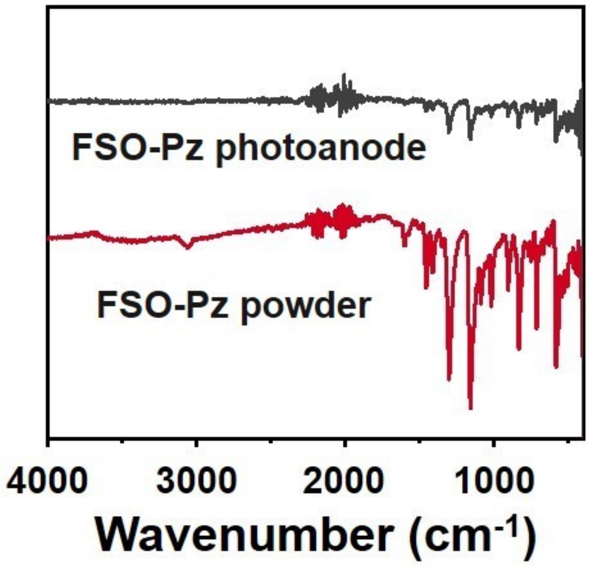

Fig. S15 FTIR spectra of FSO-Pz photoanode and powder.

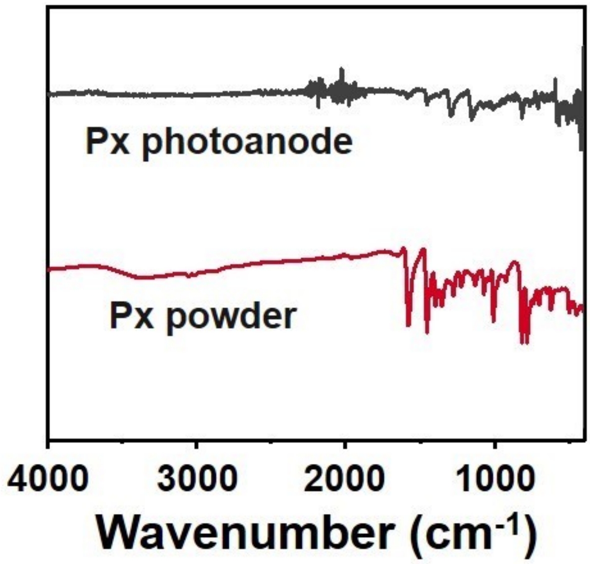

Fig. S16 FTIR spectra of Px photoanode and powder. Owing the films is thin with low quantity, and signle of Px photoanode presented similar pattern but much weaker intensity of the signals.

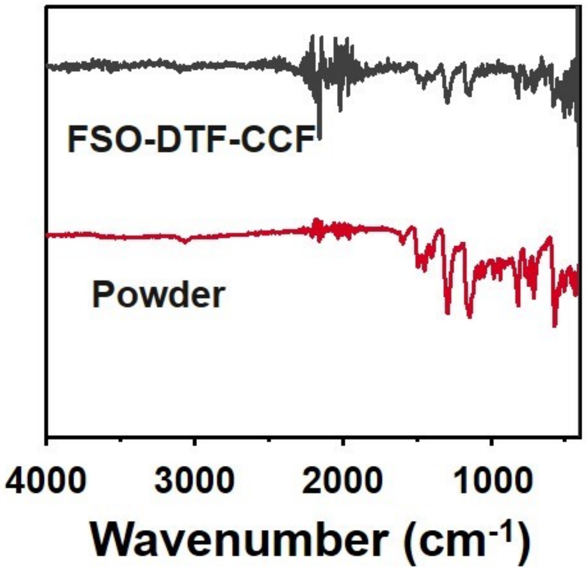

Fig. S17 FTIR spectra of FSO-DTF photoanode and powder.

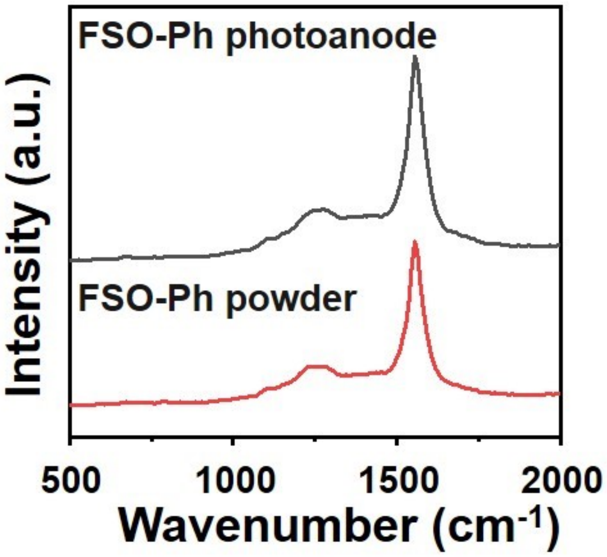

Fig. S18 Raman profiles of a FSO-Ph photoanode and FSO-Ph powder.

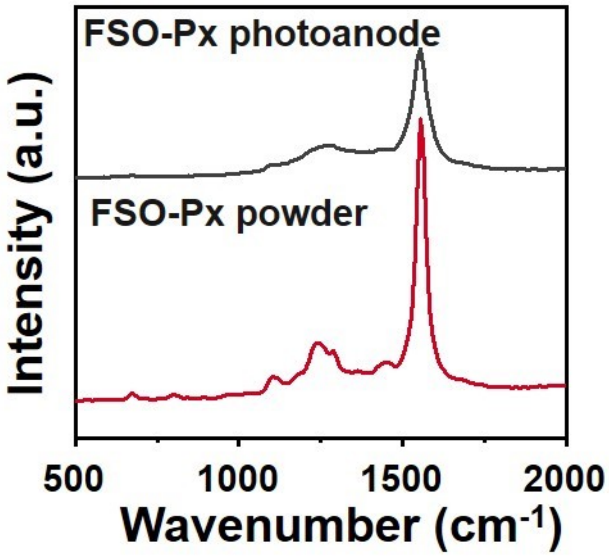

Fig. S19 Raman profiles of a FSO-Px photoanode and FSO-Px powder.

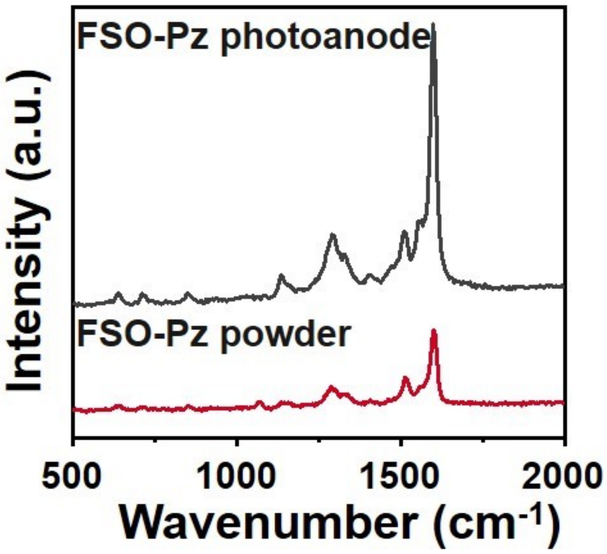

Fig. S20 Raman profiles of a FSO-Pz photoanode and FSO-Pz powder.

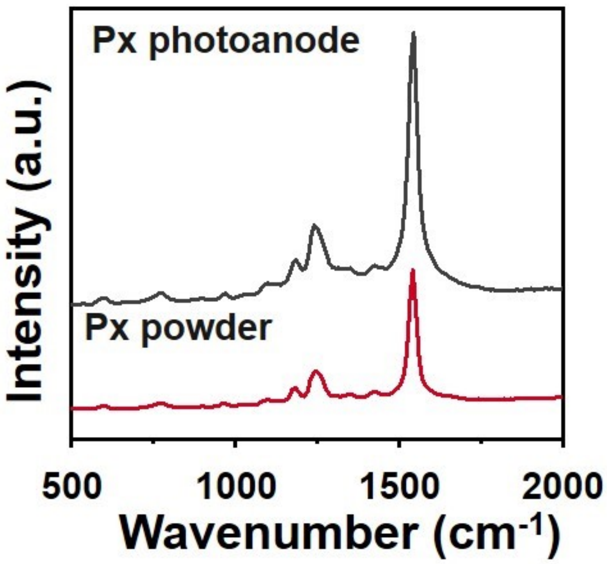

Fig. S21 Raman profiles of a Px photoanode and Px powder.

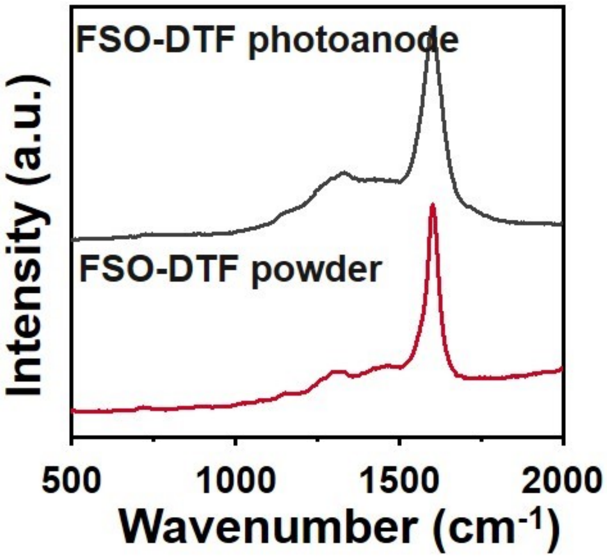

Fig. S22 Raman profiles of a FSO-DTF photoanode and FSO-DTF powder.

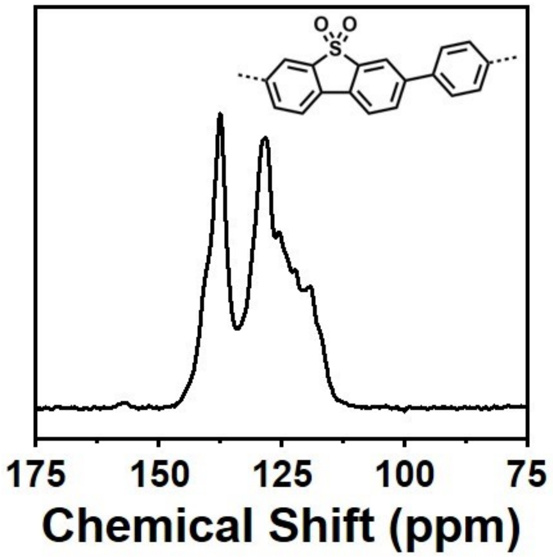

Fig. S23 ^{13}C CP/MAS NMR spectrum of FSO-Ph powder collected by peeling off the film from the photoanode.

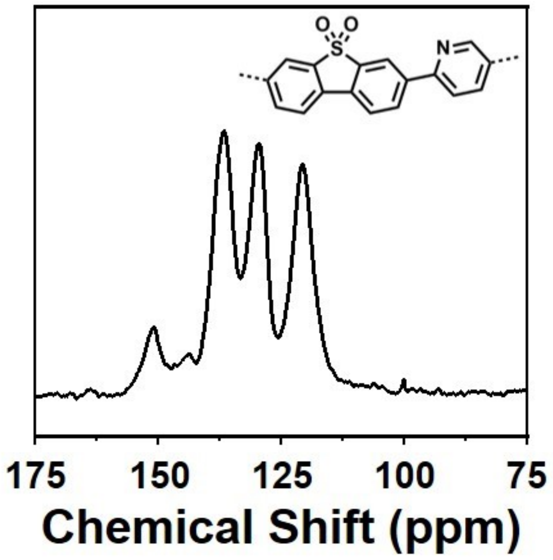

Fig. S24 ^{13}C CP/MAS NMR result of FSO-Px powder collected by peeling off the film from the photoanode.

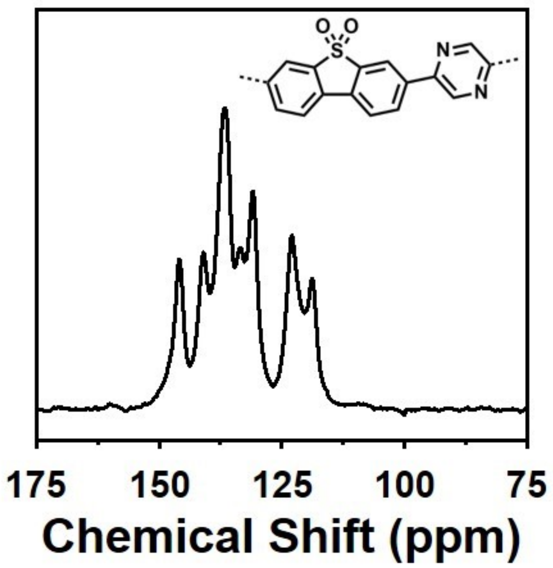

Fig. S25 ^{13}C CP/MAS NMR result of FSO-Pz powder collected by peeling off the film from the photoanode.

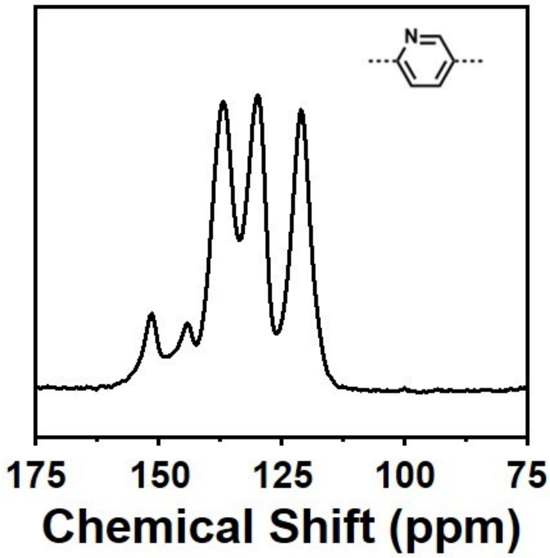

Fig. S26 ^{13}C CP/MAS NMR result of Px powder collected by peeling off the film from the photoanode.

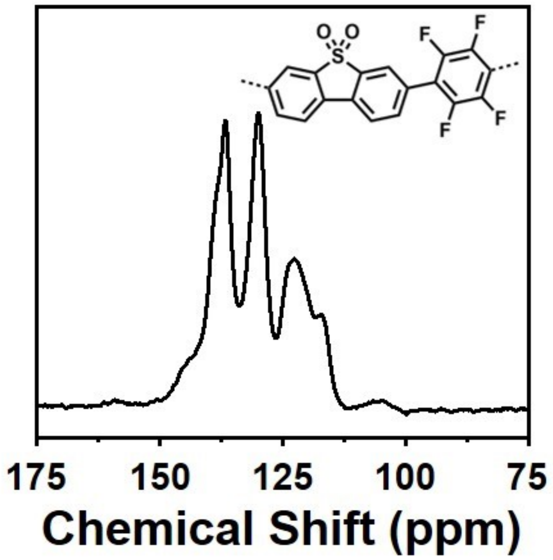

Fig. S27 ^{13}C CP/MAS NMR result of FSO-DTF powder collected by peeling off the film from the photoanode.

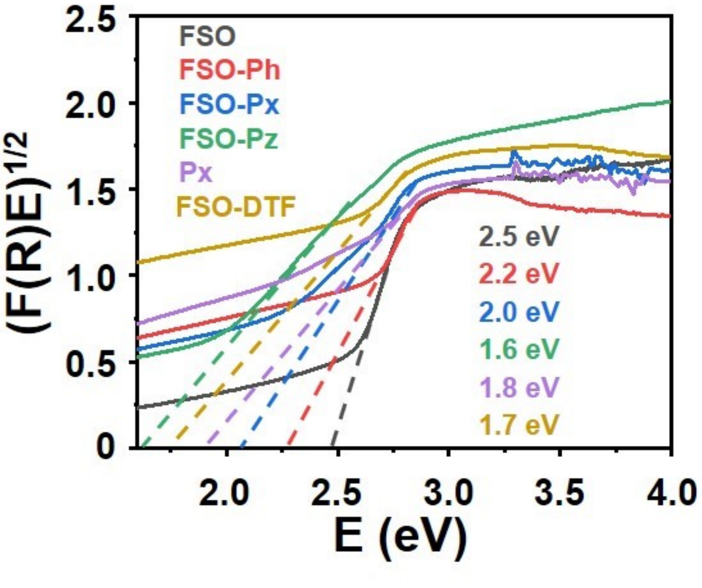

Fig. S28 Kubelka-Munk plots of FSO, FSO-Ph, FSO-Px, FSO-Pz, Px, and FSO-DTF. It's possible that the different baselines are caused by the growth directions of different polymers on the carbon cloth.

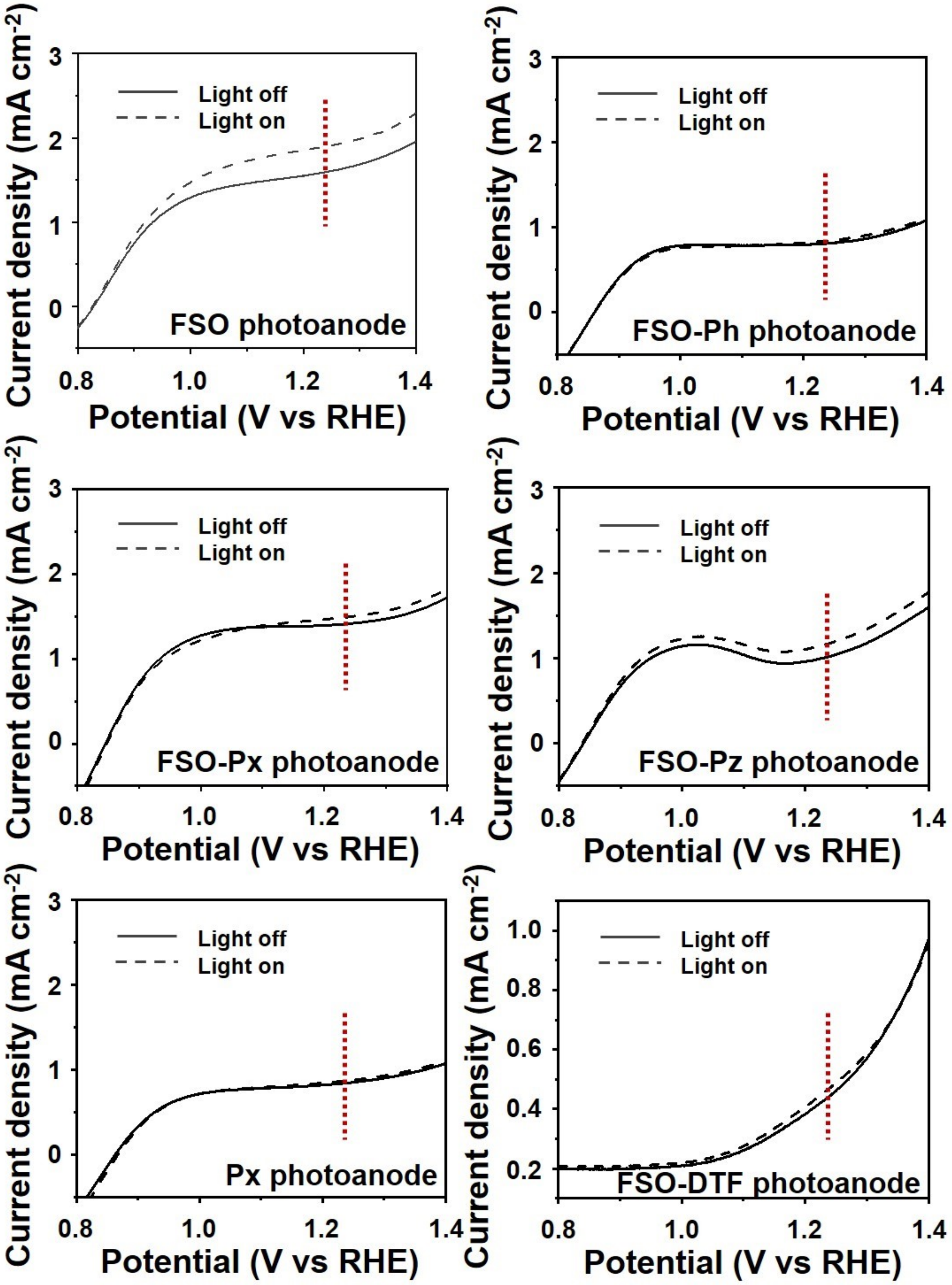

Fig. S29 Linear sweep voltammetry (LSV) spectra of FSO, FSO-Ph, FSO-Px, FSO-Pz, Px, and FSO-DTF photoanodes.

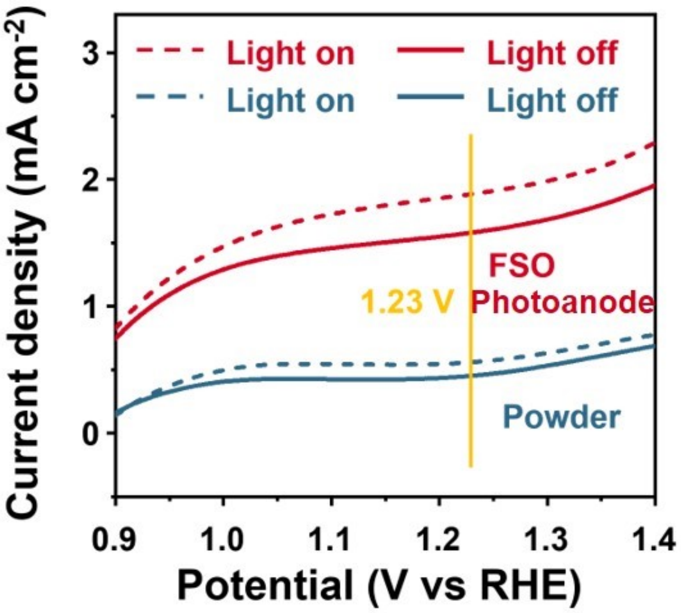

Fig. S30 LSV spectra of FSO photoanode and powder. Compared to polymer powder, the optimal photocurrent density of FSO photoanode is 140 μA cm^{-2}, which was nearly 10 times higher than FSO powder. which means that the strengthened π-π interaction connection of the polymer and CC can improve the charge dynamical obstruction and oxidizability thus improving the catalytic performance.

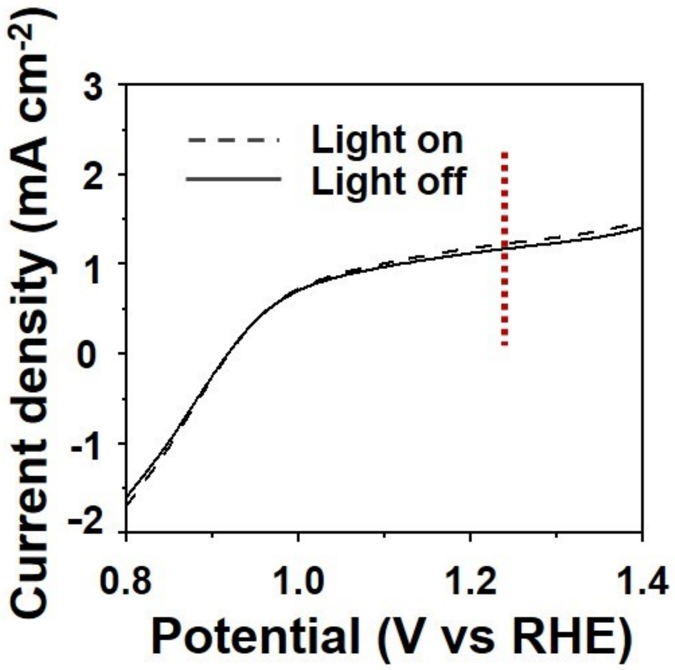

Fig. S31 LSV spectra of ^{b}FSO photoanode prepared by drop-casting.

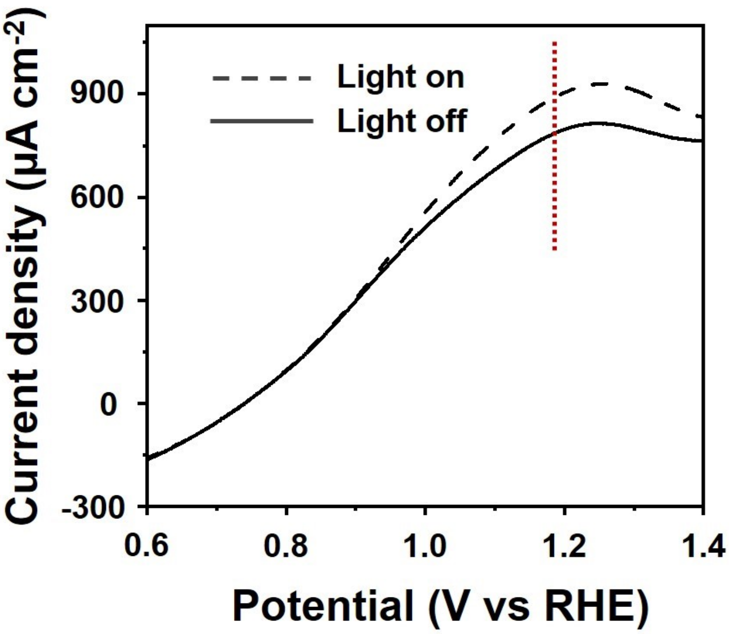

Fig. S32 LSV spectrum of ᶜFSO photoanode prepared without electrodeposition Pd.

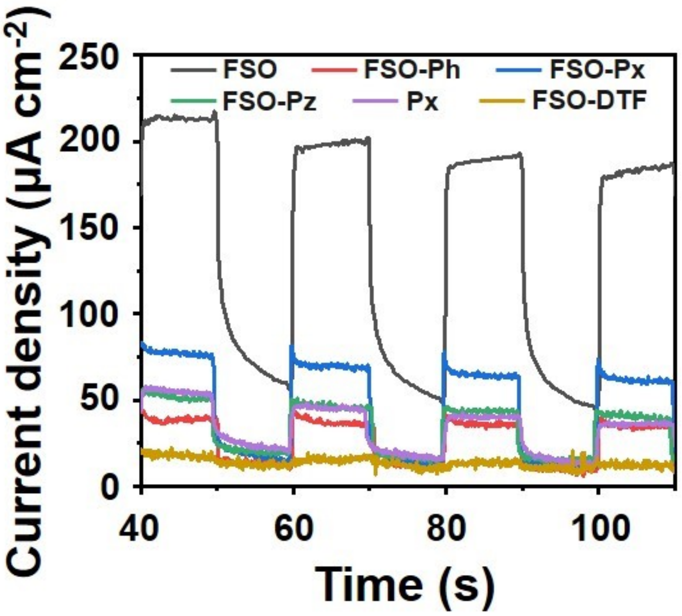

Fig. S33 Transient photocurrent responses of FSO, FSO-Ph, FSO-Px, FSO-Pz, Px, and FSO-DTF photoanodes.

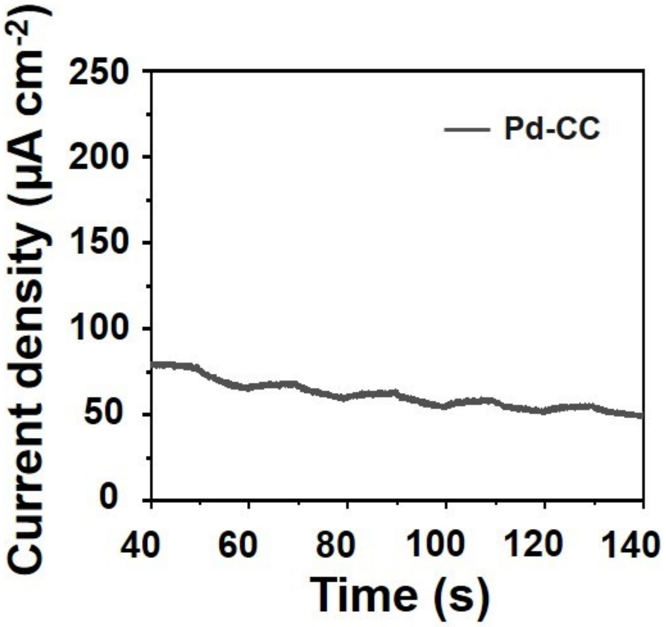

Fig. S34 Transient photocurrent responses of Pd-CC. A dark current inevitably exists in those photoanodes after electrodeposition Pd, but it does not affect the photoelectrochemical performance of those photoanodes as shown in Fig. S33.

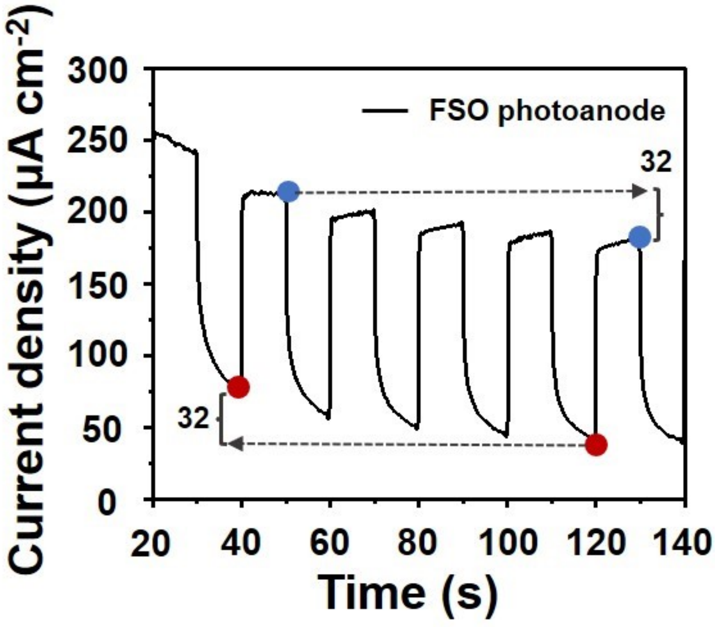

Fig. S35 Transient photocurrent responses of FSO photoanodes. The attenuation of current mainly comes from dark current, which may be due to the fact that some Pd that did not grow polymer *in-situ* did not undergo any oxidation reaction to compensate for the loss of electrons and then constant shedding from the surface of carbon cloth when applied voltage.

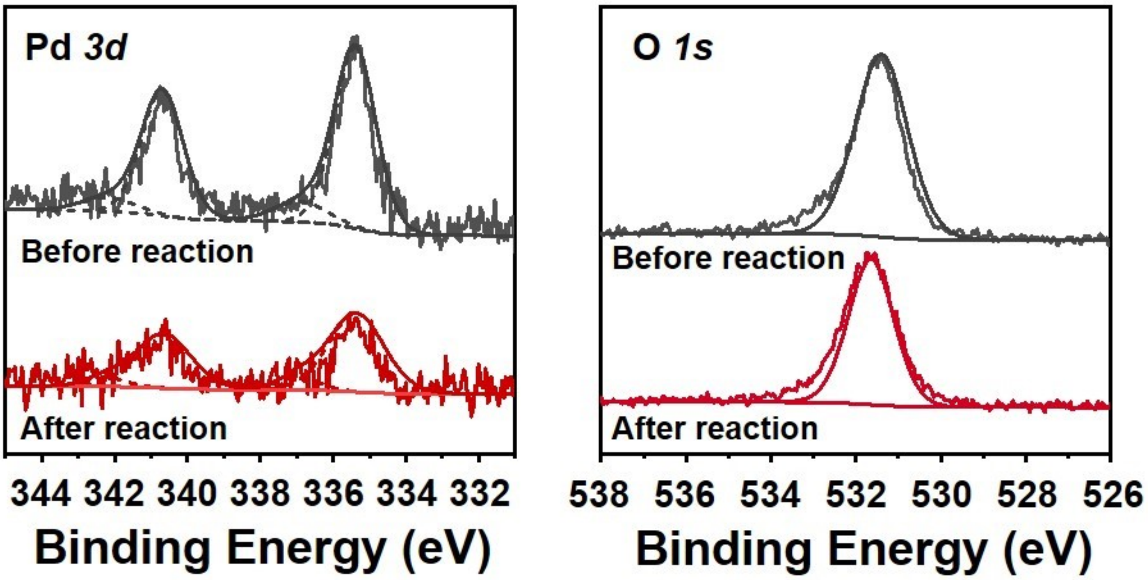

Fig. S36 Pd 3d and O 1s XPS spectra of FSO photoanode before and after reaction. The intensity of Pd 3d decreases significantly after the reaction of FSO photoanode, while O 1s remains unchanged because the Pd constant falls off.

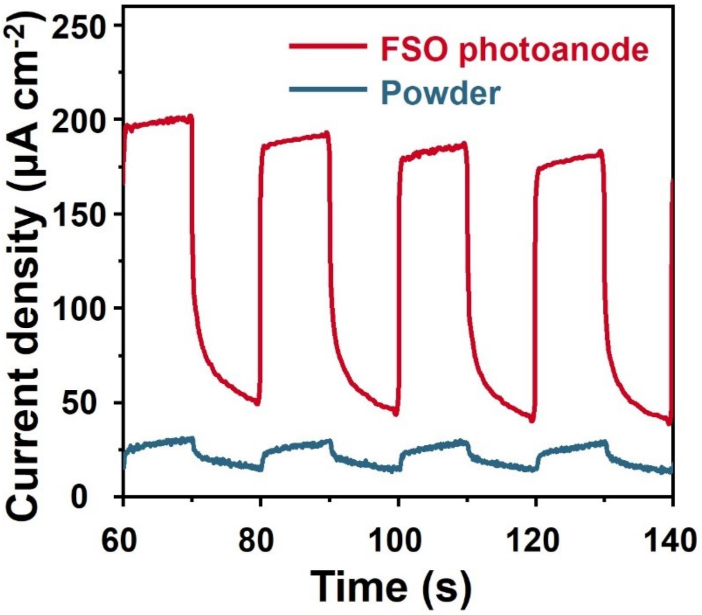

Fig. S37 Transient photocurrent responses of FSO photoanode and [a]FSO powder.

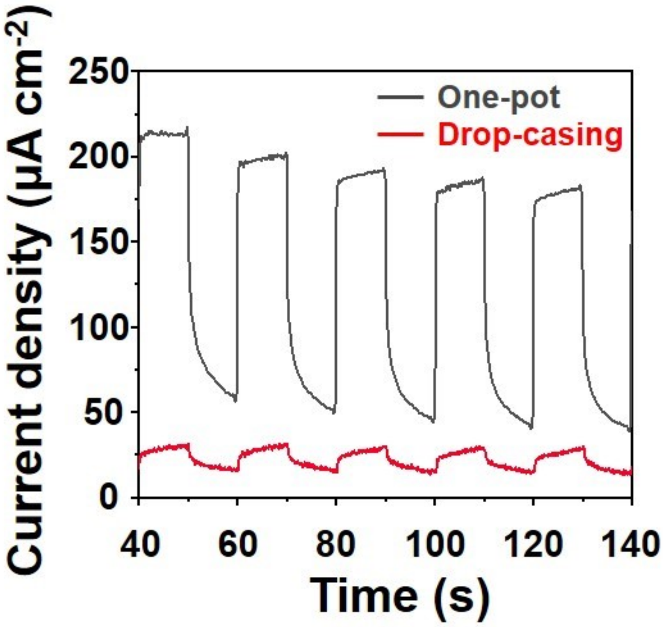

Fig. S38 Transient photocurrent responses of FSO photoanodes by different preparation methods, one-pot method (black), drop-casing (red).

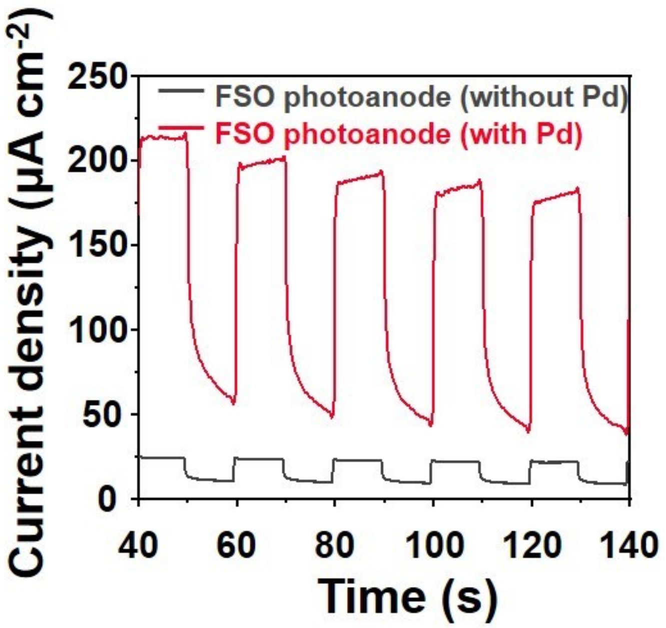

Fig. S39 Transient photocurrent responses of FSO photoanode with (red) and without (black) electrodeposited Pd. The transient photocurrent density of ^{c}FSO without Pd particles is 12 μA cm^{-2} at 1.23 V vs RHE, which is lower than 10 times than FSO photoanode with Pd particles. This result demonstrated that Pd particles can conjunct polymer and CC to effectively improve the ability of charge transport from polymer to CC.

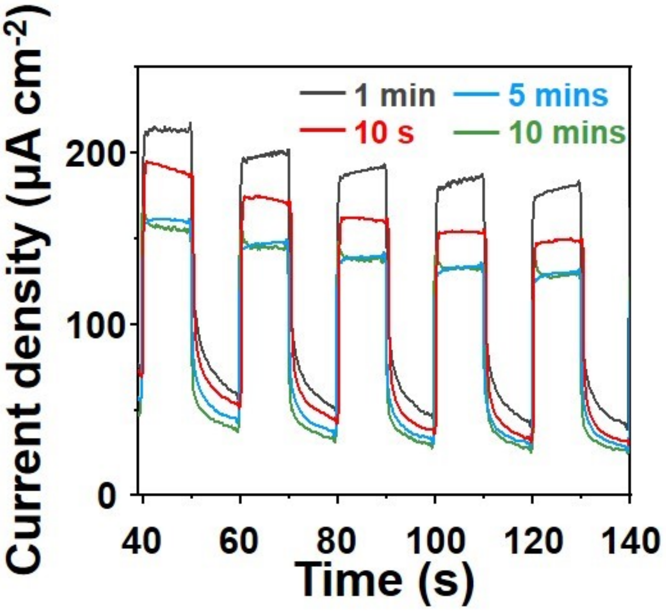

Fig. S40 Transient photocurrent responses of FSO photoanodes with different electrodeposition times.

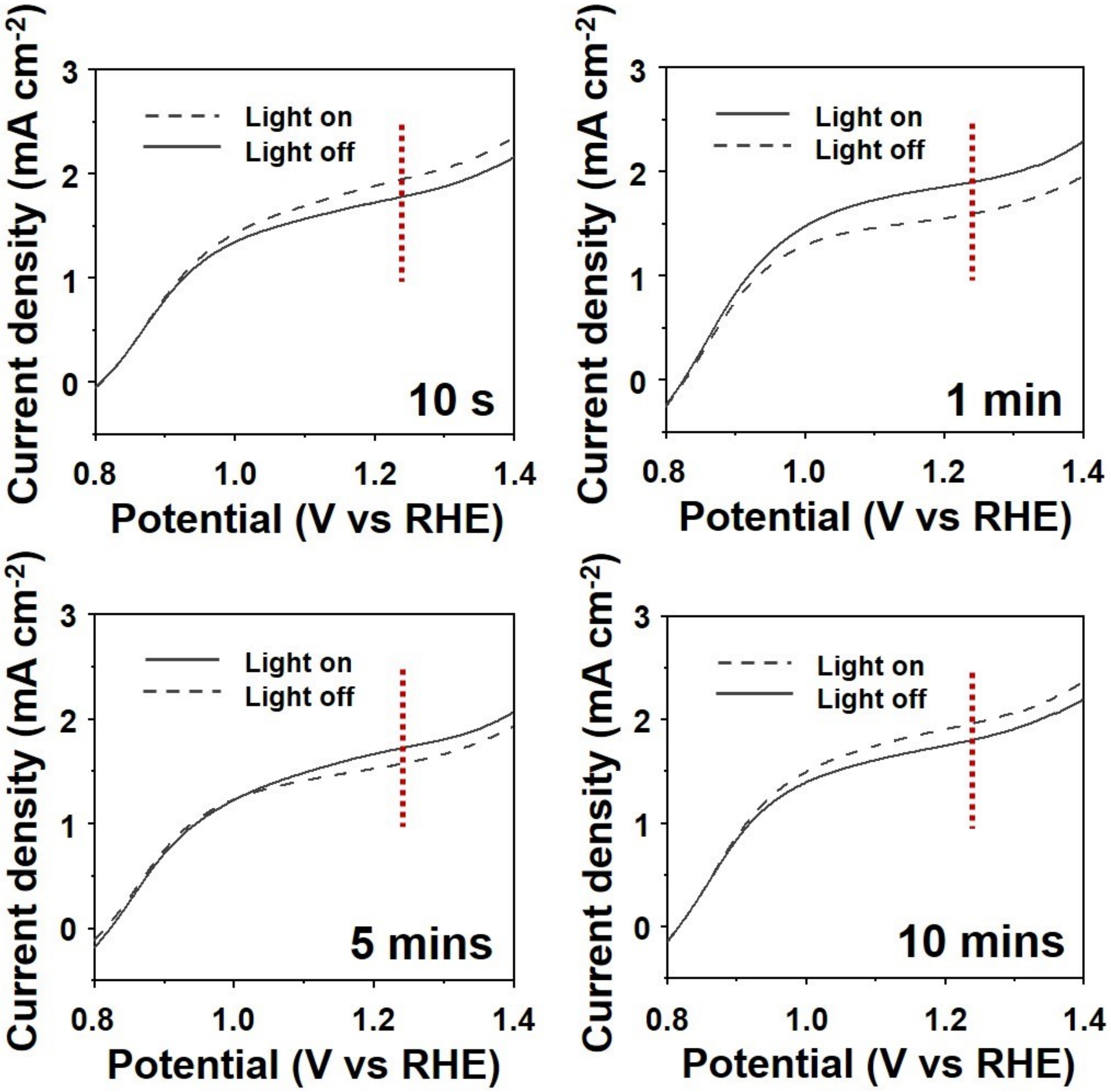

Fig. S41 LSV spectra of FSO photoanodes with different electrodeposition times. To explore the effect of different electrodeposition times on the electron-hole pairs separation ability of photoanode material, photoelectrochemical measurements were performed. LSV of all the electrodeposited films are acquired with illumination on and off by AM 1.5G, and photocurrent density dramatically increases upon 1 min electrodeposition of Pd on the CC. Compared with 10 s, 5 mins, and 10 mins, the transient photocurrent response of 1 min was present as the maximum value increased during the on-off cycles under AM 1.5G illumination. These results evidenced that a reasonable electrodeposition time is beneficial to the symmetrical distribution lamellar structure of polymer on the CC and charge transport.

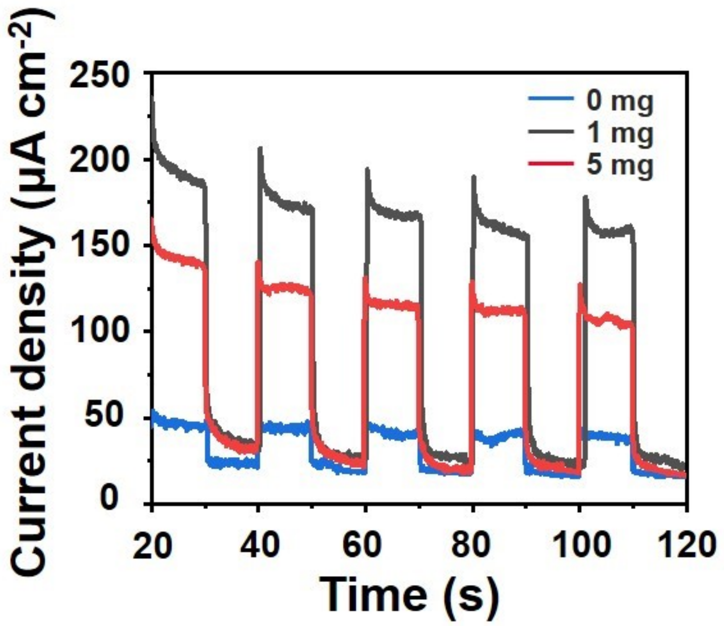

Fig. S42 Transient photocurrent response of different quantities of [Pd(PPh_3)$_4$] in Suzuki-Miyaura reaction for producing FSO photoanodes. Moderate quantities of catalysts are available to elevate the transient photocurrent response, because of the existence of competitive responses in bulk reaction and surface reaction. By contrast, when the reaction was conducted without [Pd(PPh_3)$_4$] as the catalyst, the polymer was scarcely loaded on the Pd-CC surface. A large amount of [Pd(PPh_3)$_4$] will lead to excessive competitive bulk polymerization reaction, with the polymer on the Pd-CC appearing to be block-shaped, which influences the photoelectron performance.

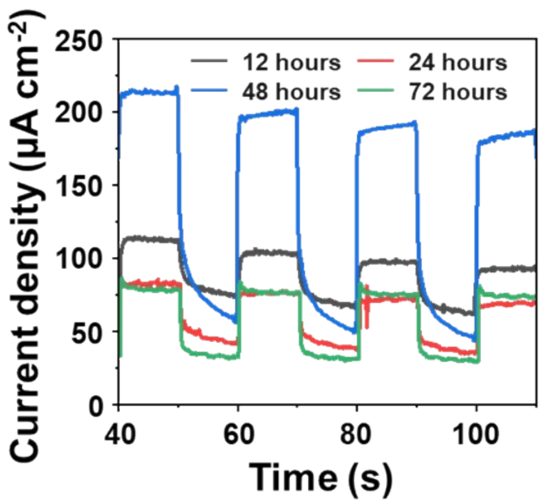

Fig. S43 Transient photocurrent response of FSO photoanodes as a function of Suzuki-Miyaura reaction time, and the corresponding structure of the FSO films were presented in Fig. S12. Increasing film thickness generally led to higher photocurrent density, peaking at a reaction

time of 48 hours, and 72 hours for the reaction time resulted bulky structure, exhibiting reduced performance. Efficient charge transfer in this system is crucial, but the length of the linear polymer cannot exceed the minority charge diffusion length.

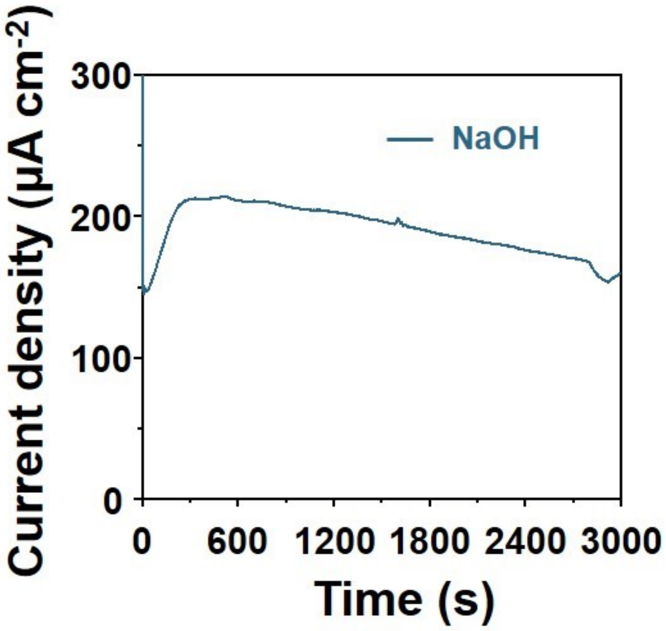

Fig. S44 Photocurrent responses of FSO photoanode for longer reaction times in NaOH.

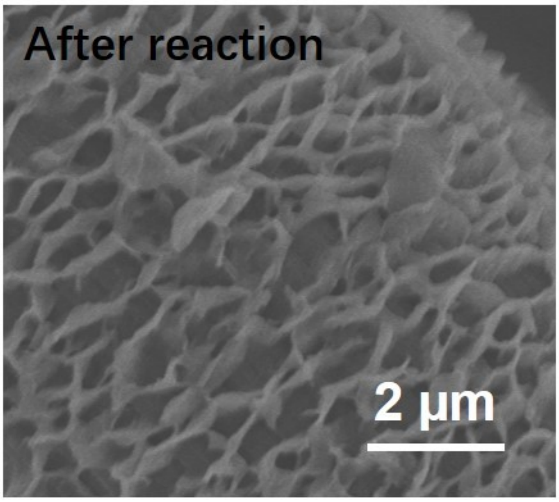

Fig. S45 SEM image of FSO photoanode after a long time experiment.

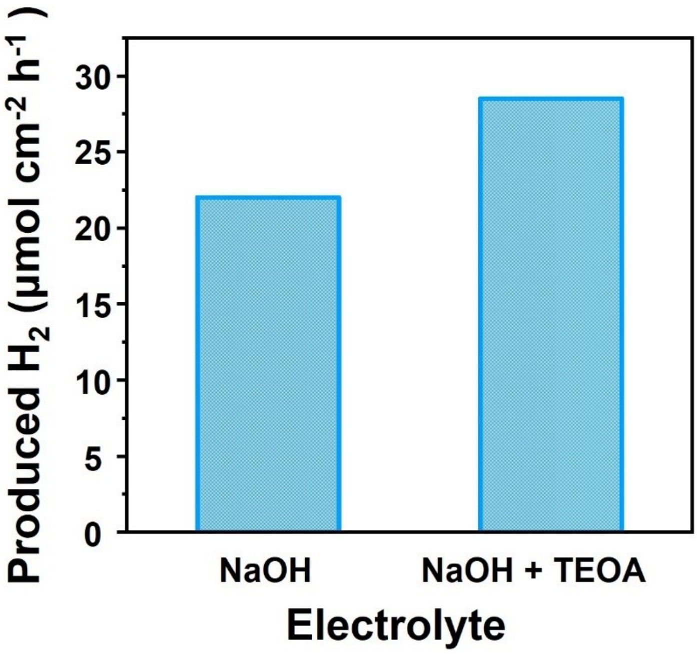

Fig. S46 The quantity of hydrogen production using FSO as photoanode under 1.0 M NaOH electrolyte with sacrificial and without sacrificial.

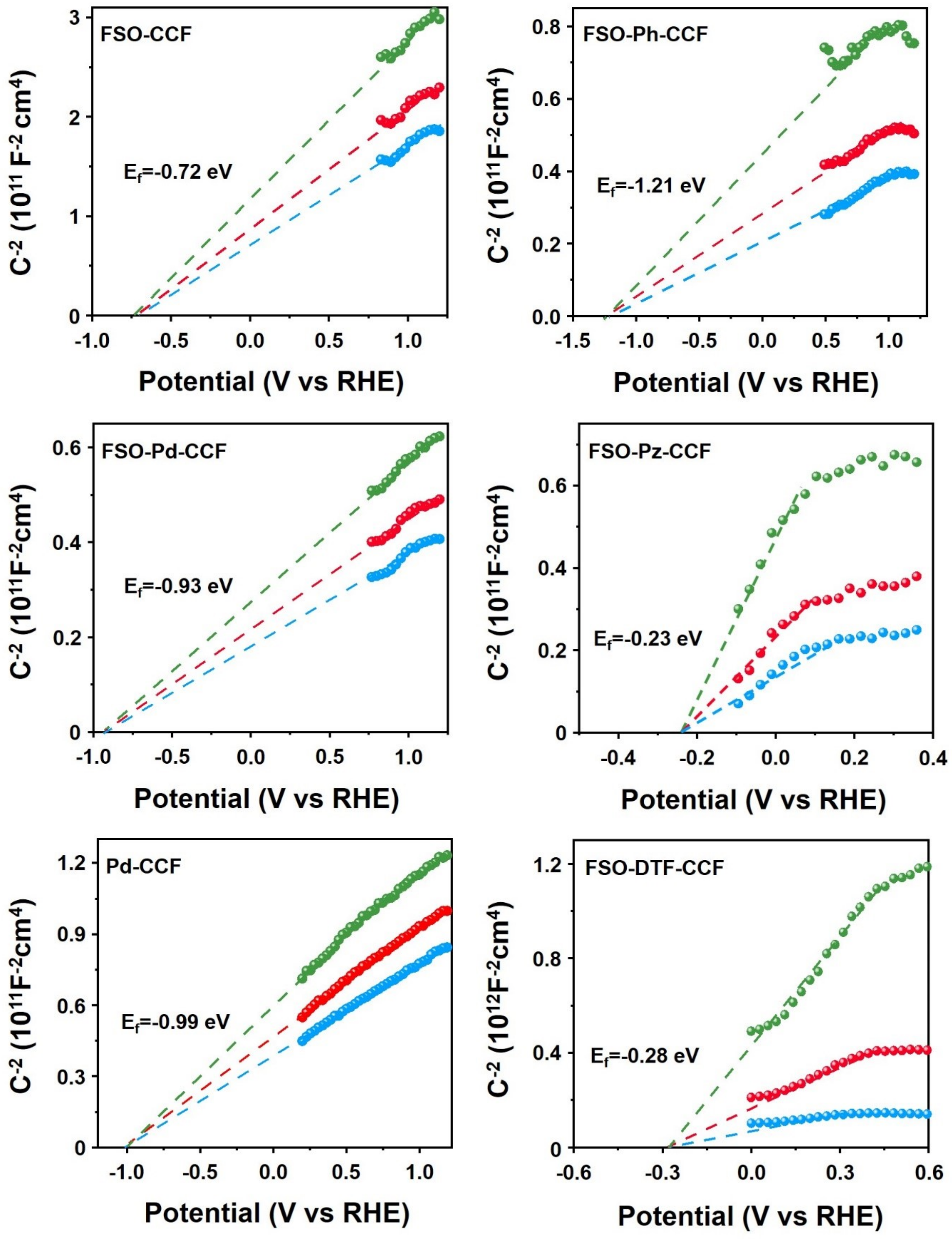

Fig. S47 Mott–Schottky plots of FSO, FSO-Ph, FSO-Px, FSO-Pz, Px, and FSO-DTF photoanodes.

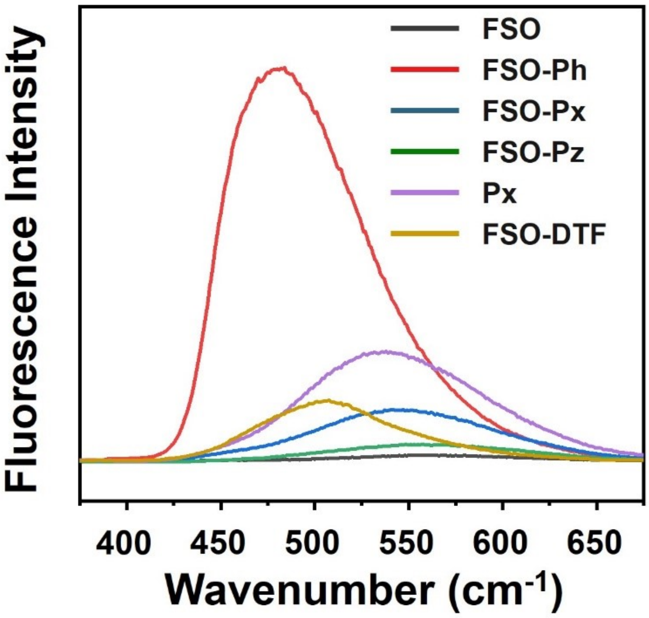

Fig. S48 Photoluminescence (PL) spectra of FSO, FSO-Ph, FSO-Px, FSO-Pz, Px, and FSO-DTF photoanodes.

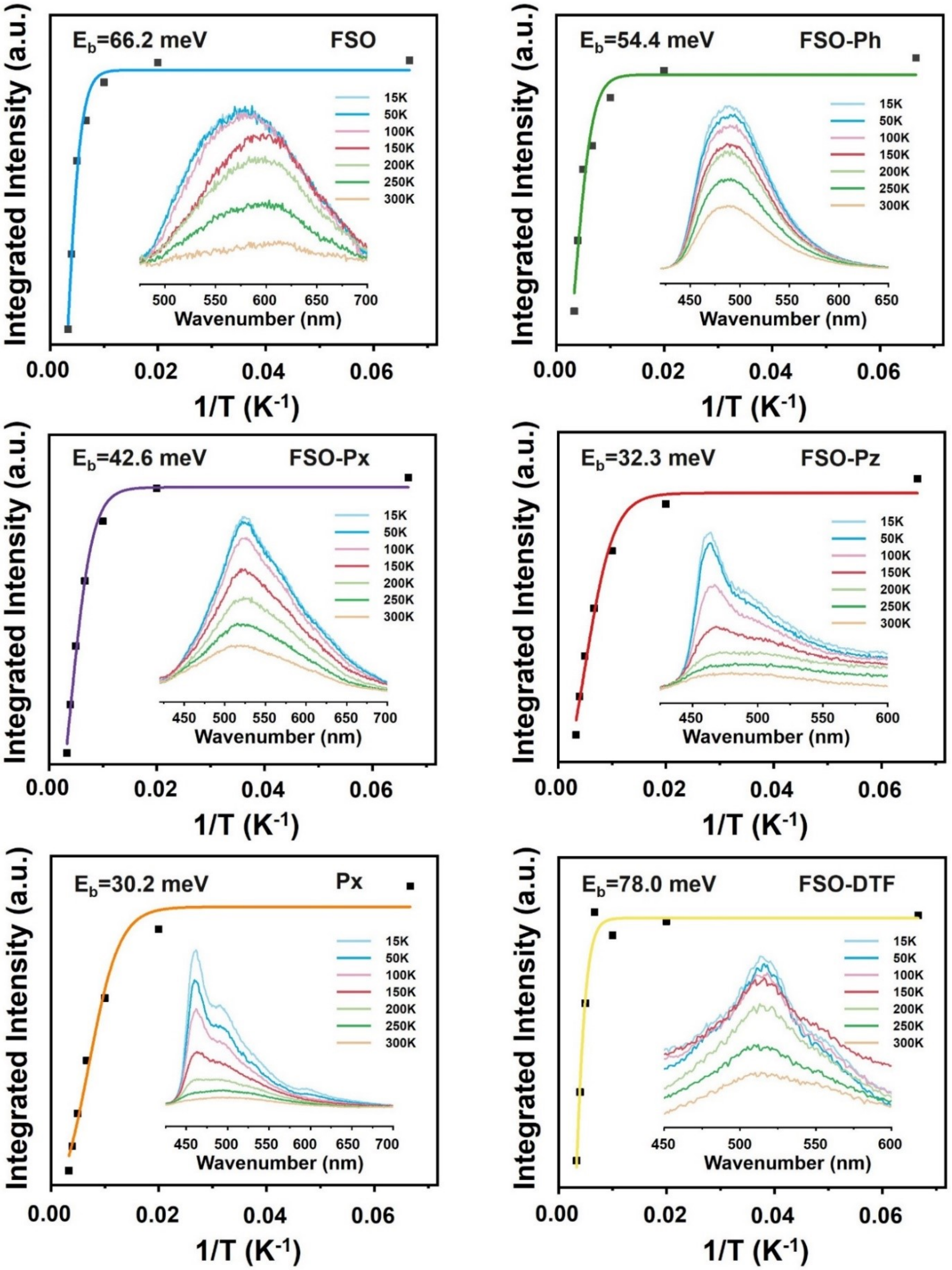

Fig. S49 Temperature dependent photoluminescence (PL) spectra of FSO, FSO-Ph, FSO-Px, FSO-Pz, Px, and FSO-DTF photoanodes.

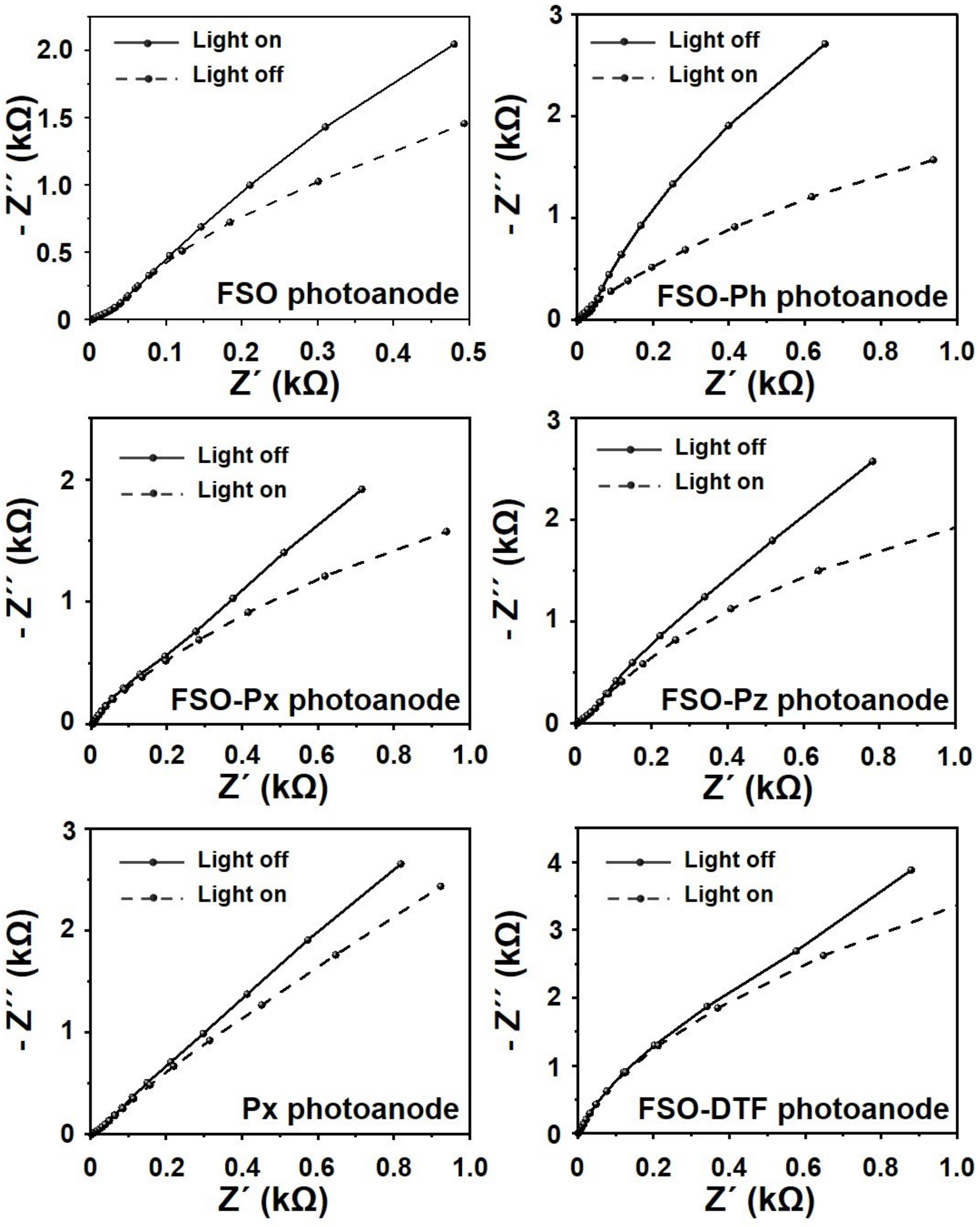

Fig. S50 Electrochemical impedance spectroscopy (EIS) spectra of FSO, FSO-Ph, FSO-Px, FSO-Pz, Px, and FSO-DTF photoanodes.

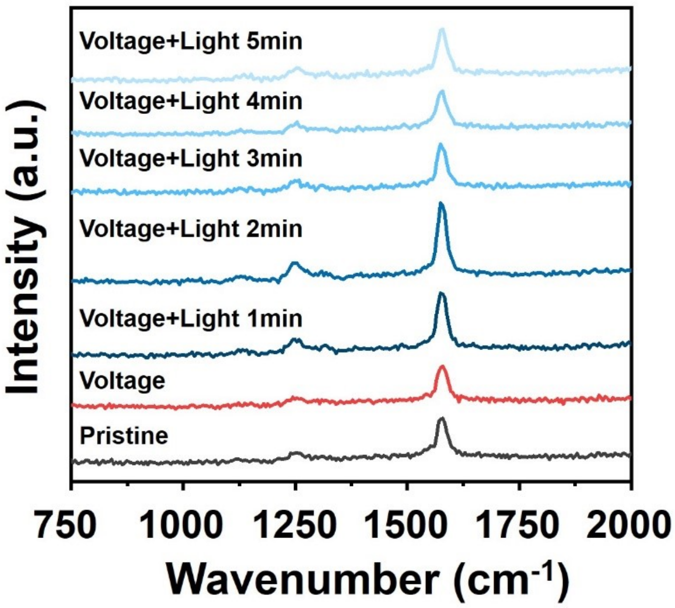

Fig. S51 *In situ* Raman spectra of the FSO photoanode for water oxidation reactions.

Table. S1 The comparison of catalytic performance for FSO photoanode and other catalysts.

Oxidation reaction	Photoanode	Irradiation condition	potential	Time	Solution	Atmosphere	Yield (%)	Selectivity (%)	Ref.
Thioanisole	CzBSe-CMP	Blue lamp		1h	MeOH	N_2	3.9	99	5
Thioanisole	TzTz-CMP-3	Blue lamp		0.4h	MeOH	Air	71	99	6
Thioanisole	BVO/U6N	300W Xe lamp AM 1.5G cm^{-2}		6h	MeOH	Air	48	99	7
Thioanisole	BVO/U6N	300W Xe lamp AM 1.5G cm^{-2}		6h	MeOH	N_2	0	99	7
Thioanisole	Zr_{12}-NBC	Blue lamp		6h	MeOH	Air	65	100	8
Thioanisole	PPET3-N2	White LED light 3.8 mW/cm^2		2h	MeOH	O_2	20	99	9
Thioanisole	BDP2	150 W quartz–halogen lamp (500 nm cutoff filter)		24h	MeOH	Air	30		10
Thioanisole	NDC2	420 LED		20h	CH_3CN	O_2	32	99	11
Thioanisole	FSO	495 nm LED	1.5 V vs Ag/AgCl	1h	H_2O	Ar	46	99	This work
Benzylamine	$BiVO_4$	460 nm LED		24h		O_2	23		12
Benzylamine	Ni3Fe-MOF-OH		1.4V vs RHE	3h	KCl(TEMPO)	Air	>90	>90	13
Benzylamine	PI	420 nm		11h	CH_3CN	Air	75	99	14
Benzylamine	TFPT-BMTH	454 nm		24h	H_2O	Air	>90		15
Benzylamine	FSO	495 nm LED	1.5 V vs Ag/AgCl	1h	H_2O	Ar	89	92	This work

Table. S2 The comparison of catalytic water oxidation performance for FSO photoanode and other catalysts.

Photoanode	Irradiation condition	Potential as RHE (V)	Solution	Photocurrent density ($\mu A\ cm^{-2}$)	Ref.
s-BCN(4%)	365 UV light	1.23	0.1 M Na_2SO_4	103.2	[16]
g-CN	AM 1.5 G	1.55	0.2 M Na_2SO_4	30	[17]
Melon	AM 1.5 G	1.23	1 M NaOH	40	[18]
$Cu_2V_2O_7$	AM 1.5 G	1.23	0.3 M K_2SO_4 and 0.2 M phosphate buffer	50	[19]
$T(Au_{25}P)_8$	300 W Xe lamp	1.0	0.5 M Na_2SO_4	59	[20]
BBL	450W Xe lamp	1.23	0.5 M Na_2SO_4	28	[21]
H-ZIS/g-C_3N_4	100 W halogen lamp	0.8 vs Ag/AgCl	0.5 M Na_2SO_4	110	[22]
FSO	AM 1.5 G	1.23	1 M NaOH	120	This work

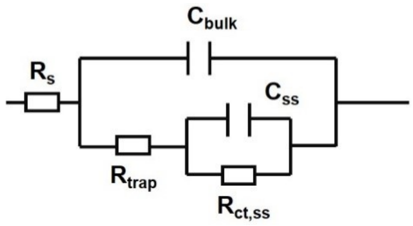

Table. S3 The fitting of the impedance spectrum irradiation with AM 1.5G of different polymer photoanodes.

Samples	R_s (Ω)	$R_{ct,ss}$ (Ω)	C_{bulk} (F)	R_{trap} (Ω)	C_{ss} (F)
FSO	3.93	5301	25.1×10^{-5}	85.1	43.8×10^{-5}
FSO-Ph	4.84	4581	15.5×10^{-5}	245.2	33.7×10^{-5}
FSO-Px	3.49	5027	21.1×10^{-5}	209.7	24.1×10^{-5}
FSO-Pz	4.45	5791	13.6×10^{-5}	168.3	20.5×10^{-5}
Px	4.91	5238	15.7×10^{-5}	93.3	30.9×10^{-5}
FSO-DTF	1.83	8594	14.3×10^{-5}	3.78	24.5×10^{-6}

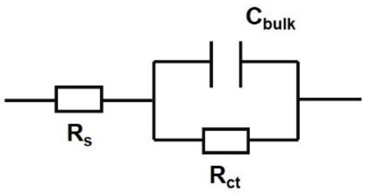

Table. S4 The fitting of the impedance spectrum of different polymer photoanodes.

Samples	$R_s(\Omega)$	$R_{ct}(\Omega)$	$C_{bulk}(F)$
FSO	3.99	6191	37.9×10^{-5}
FSO-Ph	4.08	6105	31.1×10^{-5}
FSO-Px	3.53	6797	38.8×10^{-5}
FSO-Pz	4.78	5816	46.3×10^{-5}
Pd	4.69	4733	34.9×10^{-5}
FSO-DTF	1.96	12669	32.5×10^{-5}

References

1 K.-H. Lee, K. Morino, A. Sudo, and T. Endo, *Polym. Bull.* **2010**, *67*, 227-236.

2 K. C. Moss, K. N. Bourdakos, V. Bhalla, K. T. Kamtekar, M. R. Bryce, M. A. Fox, H. L. Vaughan, F. B. Dias, and A. P. Monkman, *J. Org. Chem.* **2010**, *75*, 6771-6781.

3 R. Wazalwar, A.S. Raichur, *Thermochimica Acta* 697 (2021) 178857.

4 M. Gineys, R. Benoit, N. Cohaut, F.Beguin, S. Delpeux-Ouldriane, *Applied Surface Science* 370 (2016) 522–527.

5 Y. Zhi, Z. Yao, W. Jiang, H. Xia, Z. Shi, Y. Mu, and X. Liu, *ACS Appl. Mater. Interfaces* **2019**, *11*, 37578-37585.

6 X. Li, X. Ma, F. Zhang, X. Dong, and X. Lang, *Appl. Catal. B* **2021**, *298*, 120514-120523.

7 X. Sun, J. Liu, H. Wang, Q. Li, J. Zhou, P. Li, K. and Hu, C. Wang, B. Jiang, *Chem Eng J* **2023**, *472*, 144750-144760.

8 X.-N. Zou, D. Zhang, T.-X. Luan, Q. Li, L. Li, P.-Z. Li, and Y. Zhao, *ACS Appl. Mater* **2021**, *13*, 20137-20144.

9 J. Li, Z. An, J. Sun, C. Tan, D. Gao, Y. Tan, and Y. Jiang, *ACS Appl. Mater. Interfaces* **2020**, *12*, 35475-35481.

10 M. Liras, M. Iglesias, and F. Sánchez, *Macromolecules* **2016**, *49*, 1666-1673.

11 Y. Wang, Y. Fang, Y. Wang, H. Wu, M. Anpo, J. C. Yu, and X. Wang, *Angew. Chem. Int. Ed.* **2023**, *62*, e202307236.

12 A. D. Proctor, S. Panuganti, and B. M. Bartlett, *ChemComm* **2018**, *54*, 1101-1104.

13 W. Qiao, I. Waseem, G. Shang, D. Wang, Y. Li, F. Besenbacher, H. Niemantsverdriet, C. Yan, and R. Su, *ACS Catal.* **2021**, *11*, 13510-13518.

14 X. Wang, C. Wang, L.-H. Chen, H.-Q. Tan, Y.-M. Xing, H.-Y. Sun, Y.-N. Zhao, and D.-E. Zhang, *New J. Chem.* **2023**, *47*, 2821-2831.

15 Z. Liu, Q. Su, P. Ju, X. Li, G. Li, Q. Wu, and B. Yang, *ChemComm* **2020**, *56*, 766-769.

16 Q. Ruan, W. Luo, J. Xie, Y. Wang, X. Liu, Z. Bai, C. Carmalt, and J. Tang, *Angew. Chem. Int. Ed.* **2017**, *56*, 8221-8225.

17 J. Bian, Q. Li, C. Huang, J. Li, Y. Guo, M. Zaw, R.-Q. Zhang, *Nano Energy* **2015**, *15*, 353-361.

18 X. Li, X. Chen, Y. Fang, W. Lin, Y. Hou, M. Anpo, X. Fu, and X. Chen, *Chem. Sci.*, **2022**, *13*,7541.

19 A. Song, A. Chemseddine, I. Ahmet, P. Bogdanoff, D. Friedrich, F. Abdi, S. Berglund, and R. Krol, *Chem. Mater.* **2020**, *32*, 6, 2408–2419.

20 Y. Xiao, Q. Mo, G. Wu, K. Wang, X. Ge, S. Xu, J. Li, Y. Wu, and F. Xiao, *J. Mater. Chem. A*, **2023**, *11*, 2402.

21 P. Bornoz, M. S. Prevot, X. Yu, N. Guijarro, and K. Sivula, *J. Am. Chem. Soc.* **2015**, *137*, 15338−15341.

22 M. Khosya, D. Kumar, M. Faraz, N. Khare, *Advanced Powder Technology* 34 (2023) 104051.
